# Causality between multiple autoimmune disorders and migraine and its subtypes: a two-sample Mendelian randomization study

**DOI:** 10.3389/fneur.2024.1420201

**Published:** 2024-07-17

**Authors:** Rui Li, Jing Han, Guoliang Shao, Changyue Liu, Shuo Li, Mengmeng Wang, Dianhui Yang

**Affiliations:** ^1^Acupuncture and Tuina College, Shandong University of Traditional Chinese Medicine, Jinan, China; ^2^Department of Acupuncture, Affiliated Hospital of Shandong University of Traditional Chinese Medicine, Jinan, China

**Keywords:** autoimmune hyperthyroidism, Mendelian randomization (MR), migraine, systemic lupus erythematosus (SLE), single-nucleotide polymorphism (SNP)

## Abstract

**Introduction:**

Several studies have reported associations between various autoimmune diseases and migraine. Using Mendelian randomization (MR), this study aimed to evaluate the interplay between autoimmune diseases and migraine.

**Methods:**

Here, instrumental variables, exposure factors, and outcome factors for 10 common autoimmune diseases and migraine and its subtypes were screened. This screening utilized comprehensive statistics from Europe’s largest genome-wide association study and performed reverse MR analysis on positive results. The causality between autoimmune diseases and migraine was comprehensively assessed using multiple analytical methods. Additionally, sensitivity analyses, such as the horizontal diversity heterogeneity and leave-one-out method, were performed.

**Results:**

Random-effects inverse variance weighting analysis revealed a causal correlation between autoimmune hyperthyroidism and migraine (*p* = 0.0002), and this association was consistent across both migraine with aura (MA; *p* = 0.006) and migraine without aura (MO; *p* = 0.017). In addition, there was a positive causal association between systemic lupus erythematosus (SLE) and MA (*p* = 0.001) and between hypothyroidism and MO (*p* = 0.038). There is insufficient evidence to substantiate a causal link between outcomes and other autoimmune-related disorders, and reverse MR results did not reveal a causal relationship between migraines and these autoimmune disorders. The validity of the results was demonstrated by a sensitivity analysis; horizontal pleiotropy and heterogeneity were not observed.

**Discussion:**

This study observed a positive genetic association between autoimmune hyperthyroidism and migraines. In addition, SLE positively affects MA, and hypothyroidism contributes to the incidence of MO. These results have great significance for future research and prevention of migraine.

## 1 Introduction

Migraine is a neurological disorder that is usually accompanied by severe headaches, nausea, vomiting, and excessive sensitivity to stimuli such as light and sound. The disease has been shown to be one of the four major factors affecting the loss of healthy life years (HLY) from neurological disability (1). According to the 2016 Global Burden of Disease Study (2), migraine ranks as the third most prevalent disease and the second most disabling neurological disorder globally, affecting approximately 1.04 billion people worldwide. Thus, migraine significantly impacts the quality of life of affected individuals, placing a substantial burden on society and patients. Recent advances in genetic research have provided further insight into the complex pathophysiology of migraine. Grangeon et al. (3) indicated that genetic factors may play a key role in migraine susceptibility as they have identified several genetic loci associated with migraine. The identification of migraine-associated genes opens new avenues for personalized therapeutic interventions and a better understanding of the underlying mechanisms of migraine pathophysiology.

Immunological changes in primary headaches, particularly migraine, have been reported (4), suggesting a predisposition of some patients to immune and autoimmune diseases. Therefore, the interrelationship between autoimmune diseases and migraine has received increasing attention due to underlying common pathophysiological mechanisms, including inflammatory processes and genetic predisposition. In certain cases, the pathogenesis of autoimmune diseases seems to contribute to the onset of headaches (5, 6). In 2013, a genome-wide association study (GWAS) identified novel susceptibility genes for migraine, with several genetic variants associated with migraine susceptibility and immune system function, suggesting that genetic overlap may explain clinically observed comorbidities (7). Doulberis et al. (8) indicated that migraines are associated with immune-mediated conditions like celiac disease (CD) and inflammatory bowel disease (IBD). However, studies have shown no evidence of a common genetic basis or causal relationship between migraine and IBD or CD, contradicting previous findings (9). Recent studies showed a complex relationship between systemic lupus erythematosus (SLE) and migraine, suggesting chronic inflammatory and immune dysregulation features of SLE contribute to migraine (10, 11). Similarly, a meta-analysis found a significant association between multiple sclerosis (MS) and increased migraine risk (12). Cytokine mediated inflammation may be a common underlying mechanism for both diseases (13, 14). Given the predominantly observational nature of the evidence linking migraine and autoimmune disorders, inherent limitations arise in addressing confounding and reverse causal biases, and the causality for these associations remains uncertain.

Mendelian randomization (MR) is a research methodology that utilizes genetic variations highly associated with exposure factors as instrumental variables (IVs) to deduce causal effects between exposure factors and the outcomes under investigation. Alleles segregate independently during reproduction and are randomly combined and transmitted to offspring with equal probability during hybridization, therefore, genetic variation is generally considered to be randomly distributed at conception (15). MR studies effectively mitigate the impact of reverse causality and estimates of causal confounding in the observed data. Moreover, owing to the high accuracy of single-nucleotide polymorphism (SNP) measurements, measurement errors have less impact on results (16).

Therefore, in this investigation, a two-sample bidirectional MR analysis was employed to thoroughly evaluate the potential existence of a causal link between certain autoimmune diseases [e.g., CD, IBD, ulcerative colitis (UC), SLE, primary sclerosing cholangitis (PSC), type 1 diabetes mellitus (T1DM), rheumatoid arthritis (RA), hypothyroidism, autoimmune hyperthyroidism, and ankylosing spondylitis (AS) and migraine].

## 2 Materials and methods

### 2.1 Ethical approval

This study was based on publicly available GWAS data. Therefore, additional ethical clearance was not required.

### 2.2 MR study design

**Figure 1:**
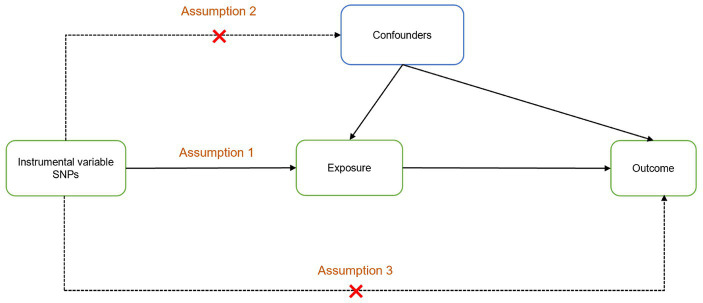
Two-sample Mendelian randomization analysis design. Assumption 1: The instrumental variables are powerfully related to exposure; Assumption 2: There is no correlation between instrumental variables and potential confounders; Assumption 3: The instrumental variables affect the outcome only through exposure.

The study design is illustrated in Figure 1. An MR design and genetic IV analysis based on pooled data were used to include SNPs as risk factors. To ensure the validity of causality estimates in MR studies, three key assumptions had to be met: (1) a robust association between genetic variation and exposure; (2) no correlation between genetic variation and potential confounders in the association between exposure and outcome; and (3) variation lacks an independent effect on outcome other than association with exposure. To satisfy these criteria, SNPs that were significantly associated with exposure on a genome-wide basis (*p* < 5 × 10^−8^) and did not have linkage disequilibrium (r^2^ < 0.001, kb = 10,000) were selected. The F-statistic was used to test the significance of the regression analysis results. The significance of the regression proof was proportional to F. It is generally accepted that the F statistic must be >10 to limit the emergence bias of weak IVs, where F = R^2^ × (N − k − 1)/[(1 − R^2^) × k] is the formula. Here, N represents the number of GWAS samples related to each immune system disorder, k denotes the number of SNPs, and R^2^ signifies the proportion of variance in immune system disorders explained by each individual SNP.

### 2.3 Data sources

#### 2.3.1 SNPs associated with autoimmune diseases

GWAS studies based on the 10 most common autoimmune diseases were selected for this study. The autoimmune diseases included were SLE, hypothyroidism, autoimmune hyperthyroidism, PSC, T1DM, RA, CD, IBD, AS, and UC. The aggregated data were obtained from the IEU OpenGWAS database.^1^ Notably, the pooled GWAS data for SLE, hypothyroidism, autoimmune hyperthyroidism, PSC, T1DM, RA, CD, IBD, AS, and UC had sample sizes of 14,267, 410,141, 17,3,938, 14,890, 17,685, 80,799, 24,269, 65,642, 22,647, and 27,432 and 7,071,163, 24,138,872, 16,380,189, 14,071,163, 7,740,245, 9,739,304, 38,037, 157,116, 99,962, and 12,255,197 SNPs, respectively. For details on all GWAS datasets, see Supplementary Table 1. The FinnGen cohorts were excluded as much as possible in order to avoid biasing the results by sample overlap and to increase the reliability of the results. Sample size and timeliness priorities were followed to select GWAS datasets for the relevant diseases from different consortia to reduce potential bias due to sample overlap. To avoid potential bias associated with race-related confounding factors, all participants included in the autoimmune diseases and migraine cohorts shared a common European ancestry.

#### 2.3.2 SNPs associated with migraine

Summary statistics for migraine and its subtypes were obtained from the FinnGen Consortium. Migraine cases were identified according to the International Classification of Diseases (ICD) codes. This process included 184,654 patients with migraine (migraine: 8,547 vs. 176,107 controls), 179,648 patients with MA (MA: 3,541 vs. 176,107 controls), and 179,322 patients with migraine and without aura (MO: 3,215 vs. 176,107 controls). All three datasets on migraine were obtained from the same study, involving more than 16 million SNPs (see Supplementary Table 1).

### 2.4 MR analysis

To assess the potential relationship between autoimmune diseases and migraine, an MR analysis using inverse variance weighting (IVW) was performed. IVW estimates represented the magnitude of the combined overall effect, which was calculated based on the Wald ratio estimates for individual SNPs. When multiple IVs are included and the three hypotheses of MR analysis are satisfied, the IVW analysis is used to integrate single effect estimates from multiple SNPs, and IVW is highly dependable and valid (17). The results obtained by IVW were reliable because it calculated a weighted average of the effect sizes of all IVs; IVW outcomes were used as the primary outcome measure in this study. Four other MR analysis methods were also used: weighted median (WME), MR-Egger, weighted mode (WM), and simple mode (SM). When effective IVs account for at least 50% of the SNPs, WME can estimate the heterogeneity of causal effects (18). The MR-Egger method assumes that the association between instrumental variables and exposure is independent of the effect of instrumental variables on the multiplicity of outcomes and considers the presence of an intercept term, which helps to provide accurate estimates of causal effects in the presence of a multiplicity of effects bias (19). MR-Egger regression can provide tests for unbalanced pleiotropy and considerable heterogeneity, while the same underexposed variants require larger sample sizes. The SM and WM are designed to derive a singular IV causal effect estimate from multiple IVs, and they possess the advantages of reduced bias and a lower probability of Type I errors (20, 21). When only one IV was included in the MR model, the Wald ratio method was used to estimate the causal effect of exposure variables on disease outcomes (22). Data were analyzed using the TwoSampleMR software package, operated in R software (version 4.3.1), to assess the strength of the relationship by odds ratio (OR). An OR > 1 indicates that exposure increased the incidence of the outcome; OR < 1 suggests that exposure decreased the incidence of the outcome; and OR = 1 indicates no relationship.

### 2.5 Analysis of sensitivity

A sensitivity analysis was performed to measure the feasibility and stability of the conclusions, and horizontal pleiotropy was determined by the intercept term of the MR-Egger regression; *p* < 0.05 suggests the presence of horizontal pleiotropy. Horizontal pleiotropy was further identified via outlier removal using MR-PRESSO (23). The *p*-value was verified by the Cochran’s Q statistic, *p* > 0.05 indicated heterogeneity (24). Sensitivity analyses were performed to assess whether individual SNPs had an impact on the relationship between exposure and outcome using the “leave-one-out” method (i.e., removing each SNP in turn and computing the cumulative effect of the remaining SNPs). Funnel and forest plots were used to visualize the results. The MR operations were analyzed using “TwoSampleMR” in the R software (version 4.3.1).

## 3 Results

### 3.1 Causality between autoimmune diseases and migraine

**Figure 2:**
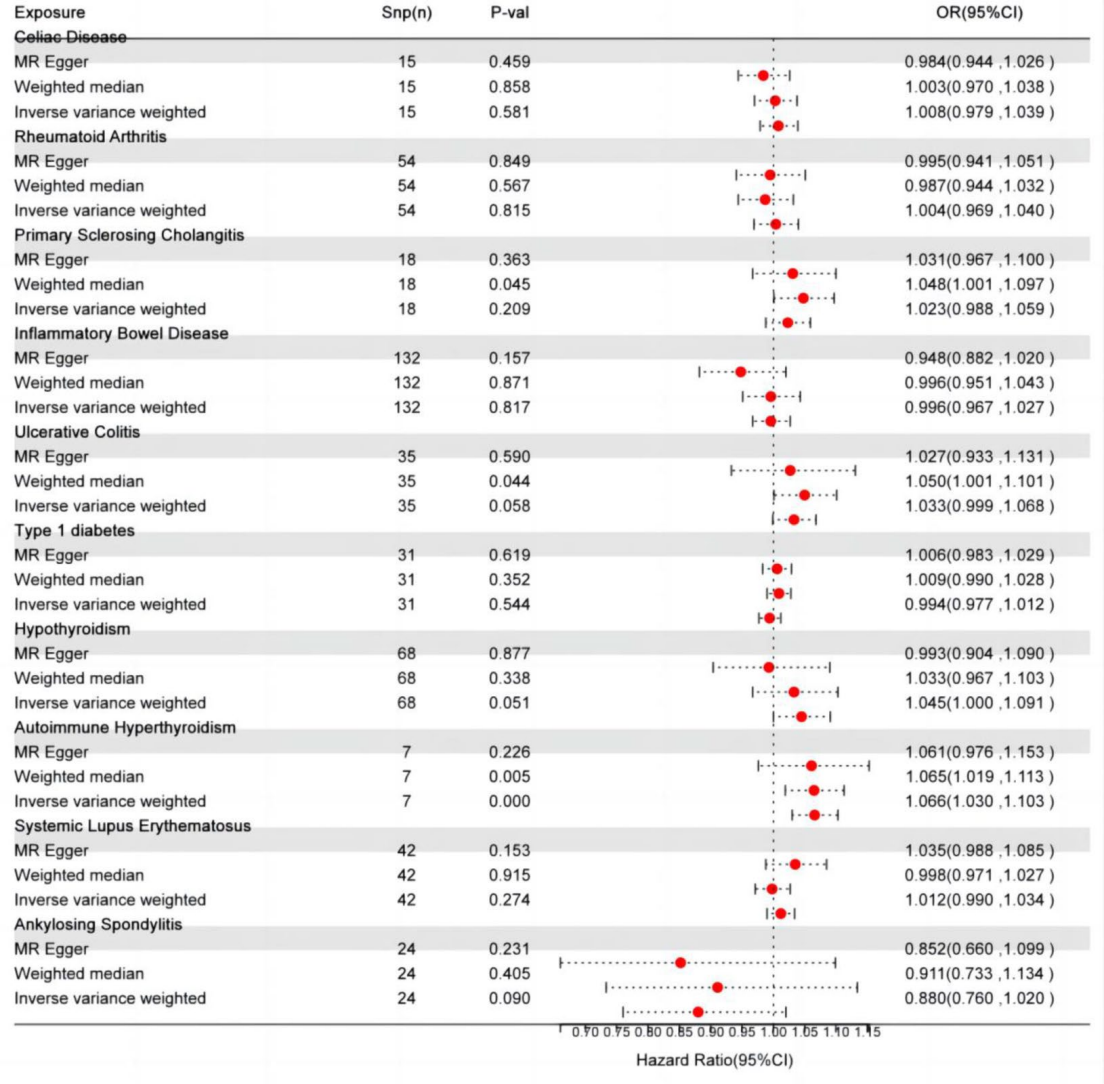
Autoimmune disease-related effects on migraine. SNP(n), number of single-nucleotide polymorphisms; OR, odds ratio; CI, confidence interval.

**Figure 3:**
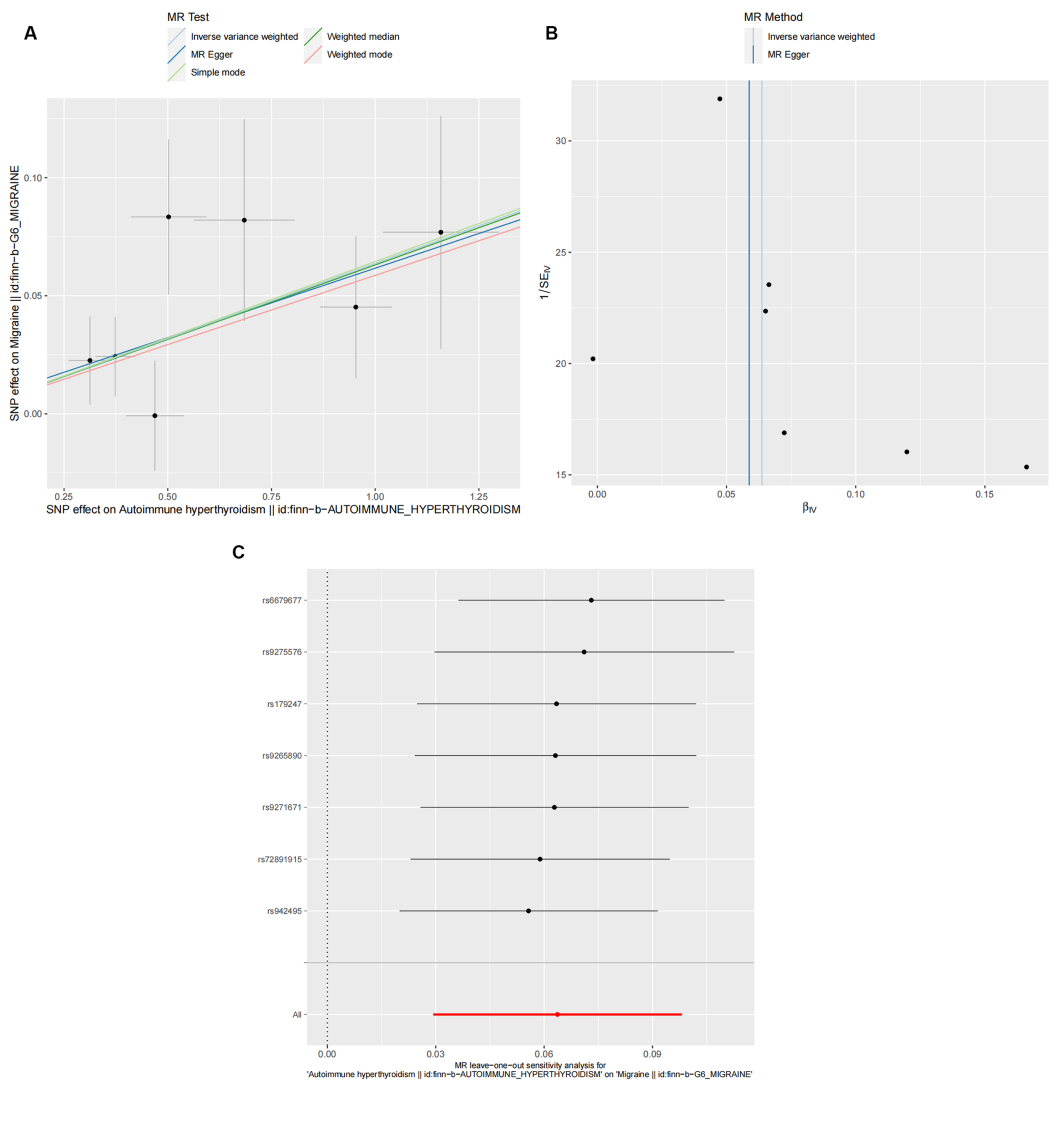
Effect of autoimmune hyperthyroidism on migraine. **(A)** Scatter plot: Analyses were conducted using the conventional inverse variance weighting and MR-Egger. **(B)** Funnel diagram: Analyses were conducted using the conventional inverse variance weighting, MR-Egger, weighted median, simple mode, and weighted mode. **(C)** Leave-one-out sensitivity test.

IVW, as the primary analysis, showed that autoimmune hypersensitivity was forward correlated with migraine (OR 95% confidence interval (CI) = 1.066 [1.030–1.103], *p* = 0.0002), supported by WM analysis (OR 95% CI = 1.065 [1.019–1.113], *p* = 0.005) providing evidence for robustness. MR-Egger regression found no evidence of horizontal pleiotropic imbalance (*p* = 0.903). Among the other MR analysis methods, WM showed a positive association between primary sclerosing cholangitis (OR 95% CI = 1.048 [1.001–1.097], *p* = 0.045), UC (OR 95% CI = 1.050 [1.001–1.101], *p* = 0.044), and migraine, whereas IVW did not exhibit a positive causal association with these diseases (Figure 2). No heterogeneity was detected in the heterogeneity test analyses (Q test or MR-Egger test). The MR-Egger intercept test revealed that the results were not multivariate. The leave-one-out methodology showed that the carry-out effect estimates remained consistent after individually removing each SNP, with no significant effect. The robustness of the MR results was verified using an additional analytical method (Figures 3A–C). Nonetheless, the association between other autoimmune diseases and migraine was not captured in IVW, and the reliability of MR affecting the estimation was demonstrated by sensitivity analysis (Supplementary Table 2).

### 3.2 Causality between autoimmune diseases and migraine subtypes

**Figure 4:**
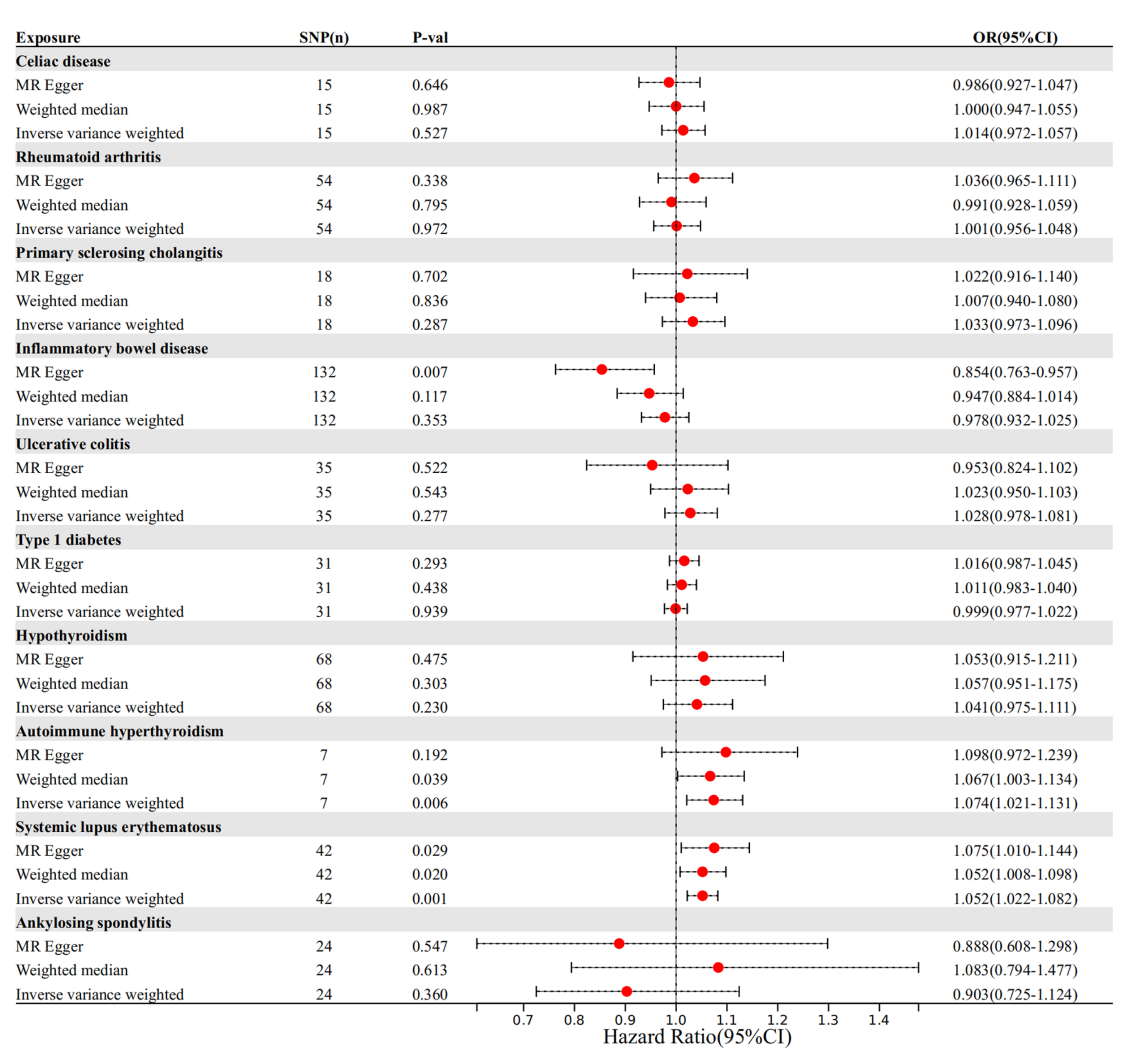
The causal impact of autoimmune disease on migraine. SNP(n), number of single-nucleotide polymorphisms; OR, odds ratio; CI, confidence interval.

**Figure 5:**
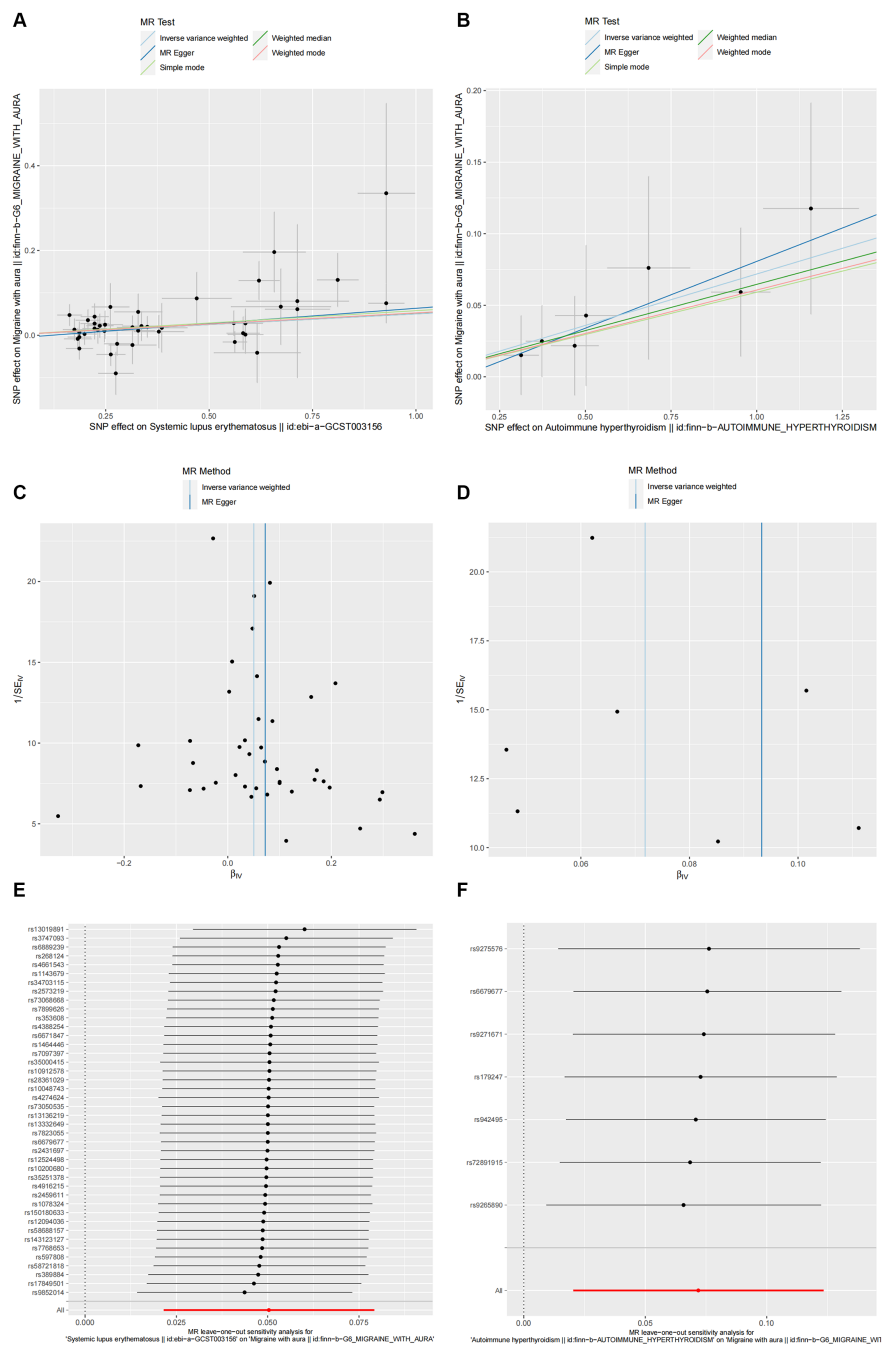
The causal link between SLE **(A,C,E)** and autoimmune hyperthyroidism **(B,D,F)** in MA. **(A,B)** Scatter plot: Analyses were conducted using the conventional inverse variance weighting and MR-Egger. **(C,D)** Funnel plot: Analyses were conducted using the conventional inverse variance weighting, MR-Egger, weighted median, simple mode, and weighted mode. **(E,F)** Leave-one-out sensitivity test.

The cause-and-effect association between autoimmune diseases and migraine is shown in Figure 4. SLE was positively associated with MA, according to the IVW results (*p* = 0.001, OR 95% CI = 1.052 [1.022–1.082]), consistent with the MR analysis methods (MR-Egger and WM) in terms of orientation and results (Figure 4), indicating that this association is robust. The IVW results showed a causative link between autoimmune hypersensitivity and MA (*p* = 0.006, OR 95% CI = 1.074 [1.021–1.131]), while the WM results (*p* = 0.039, OR 95% CI = 1.067 [1.003–1.134]) had the same orientation (Figure 4), and MR-Egger results suggested that further analysis was needed to explain this association, taking into account some bias factors. No heterogeneity was found in the heterogeneity test analyses (Q test or MR-Egger test). The MR-Egger intercept test confirmed that the results were not pleiotropic. Moreover, the robustness of the MR effect estimates was validated using other analytical methods (Figures 5A–F; Supplementary Table 3). The IVW results showed no causal relationship between IBD and MA; however, the MR-Egger method results were significant (*p* = 0.007, OR 95% CI = 0.854 [0.763–0.957]), indicating that the results did not support a causal relationship after considering the weight of multiple genetic variants. The IVW approach demonstrated no correlation between other autoimmune diseases and MA, and the reliability of MR affecting the estimation was demonstrated by sensitivity analysis (see Supplementary Table 4).

**Figure 6:**
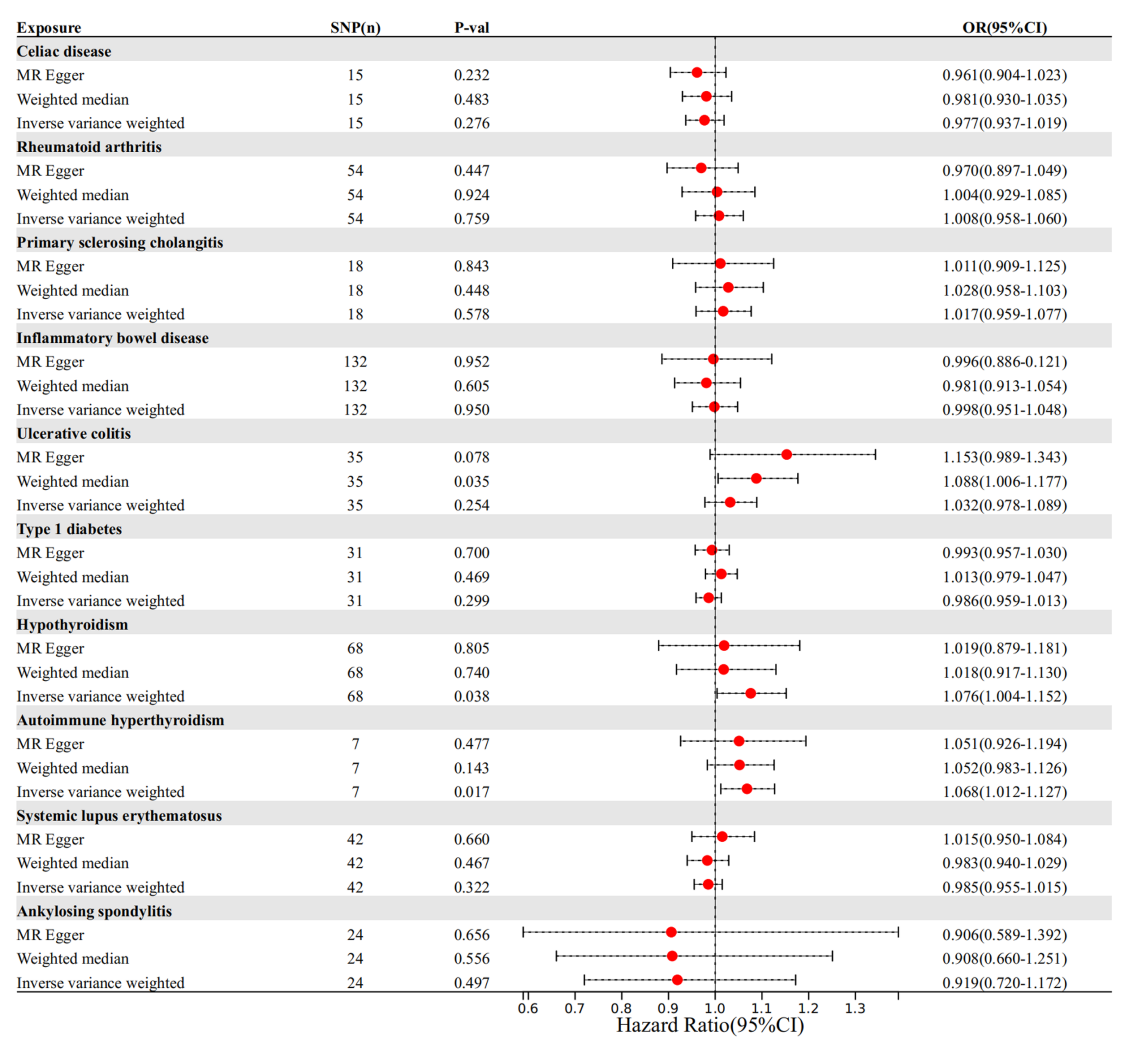
The causal link between autoimmune diseases and MO. SNP(n), number of single-nucleotide polymorphisms; OR, odds ratio; CI, confidence interval.

**Figure 7:**
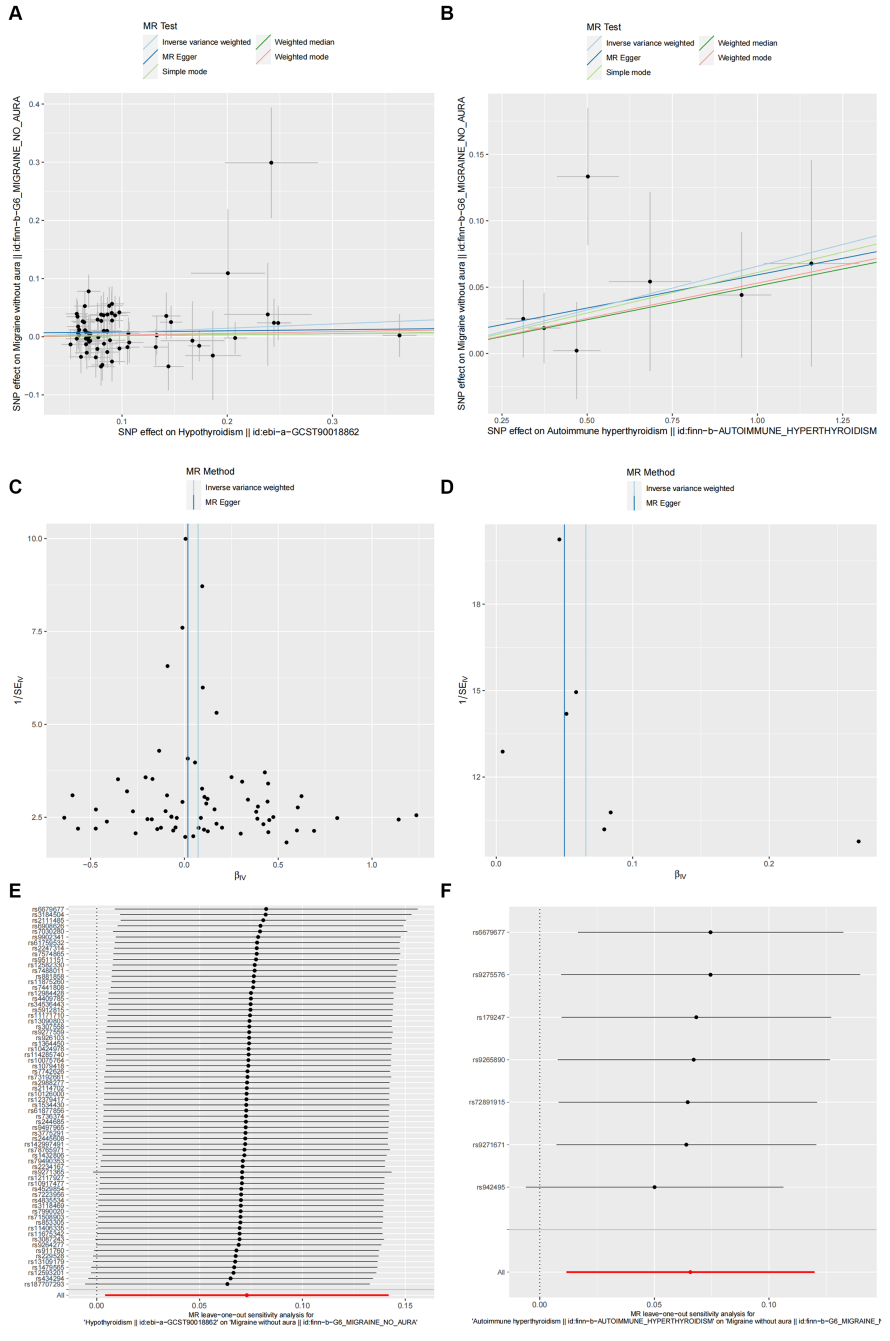
Hypothyroidism **(A,C,E)** and autoimmune hyperthyroidism **(B,D,F)** causality to MO. **(A,B)** Scatter plot: Analyses were conducted using the conventional inverse variance weighting and MR-Egger. **(C,D)** Funnel plot: Analyses were conducted using the conventional inverse variance weighting, MR-Egger, weighted median, simple mode, and weighted mode. **(E,F)** Leave-one-out sensitivity test.

According to the IVW method, two autoimmune diseases were positively and causally associated with MO, including hypothyroidism (*p* = 0.038, OR 95% CI = 1.076 [1.004–1.152]), and autoimmune hypersensitivity (*p* = 0.017, OR 95% CI = 1.068 [1.012–1.127]), consistent with the findings of the WM and MR-Egger analysis methods, indicating a stable association (Figure 6). The WM method identified a weak positive cause-and-effect relation between UC and migraine (*p* = 0.035, OR 95% CI = 1.088 [1.006–1.177]), whereas IVW did not find a causal association (*p =* 0.254, OR 95% CI = 1.032 [0.978–1.089]) (Figure 6). No heterogeneity was observed during the heterogeneity test analysis (Q-test and MR-Egger). The MR-Egger intercept test confirmed that the results were not pleiotropic. The stability of MR analysis was further supported by the leave-one-out method, scatter plot, and funnel plot (Figures 7A–F; Supplementary Table 3).

### 3.3 Causality between migraine and autoimmune diseases

To further examine the causal relationship, we performed reverse MR for autoimmune diseases with positive migraine results. During the analysis, when we set the statistical threshold to *p* < 5 × 10^−8^, the number of SNPs associated with migraine was not sufficient to support our study; therefore, the threshold was expanded to p < 5 × 10^−6^ to incorporate a sufficient number of SNPs to address the above limitation. Nevertheless, the IVW approach demonstrated no correlation between migraine and diseases of the incorporated immune system (see Supplementary Table 5).

## 4 Discussion

This study is the first to employ MR analysis to evaluate the causal relationship between multiple autoimmune disorders and migraines, including migraine, MA, and MO. These findings confirm the relationship between autoimmune hyperthyroidism and migraine, and this association is consistent between MO and MA, suggesting that autoimmune hyperthyroidism increases the likelihood of migraine (migraine, MA, and MO). Furthermore, there is a positive causal relationship between SLE and MA, and hypothyroidism has the potential to promote MO occurrence. Other immune system disorders included in the study were not related to migraines. Furthermore, our reverse MR analysis did not find any relevant relationships between migraine (migraine, MA, and MO) and autoimmune disorders.

Migraine is a common nervous system disease, and trigeminal neurovascular mechanisms play an important role in its pathogenesis. The activation of immune cells near afferent nerves (i.e., resident mast cells and macrophages) produces various mediators, such as serotonin, proinflammatory cytokines, and chemokines, all of which are closely related to the peripheral sensitization of trigeminal nerve terminals (25). Polymorphisms in genes encoding human leukocyte antigens and cytokines have been identified as potential pathogenic factors for autoimmune diseases. These genetic variations are also linked to the pathogenesis of migraine (26). Patients with migraine experience immunological changes in their bodies, which can play a role in their physiopathology, primarily by altering the levels of inflammatory cytokines in the body (27–30). Increased levels of these cytokines and chemokines during migraine attacks indicate a proinflammatory state. This suggests a strong link between autoimmune diseases and migraine. A cohort study by Nowaczewska et al. (31) suggested that Hashimoto’s thyroiditis, may cause migraine chronicity. This chronicity may be caused by the persistent inflammatory state and immune dysregulation characteristic of AIT. However, the causal relationship between migraine and autoimmune disorders remains unclear (4). The relationship between autoimmune diseases and migraines is complex and multifaceted, involving various biological pathways and molecular mechanisms. In addition to immune-mediated inflammation, genetic factors may predispose individuals to both conditions. Furthermore, shared genetic loci and pathways involved in immune regulation and neural signaling may be notable contributors. The blood–brain barrier (BBB) also plays a crucial role. BBB integrity is essential for maintaining central nervous system homeostasis. Autoimmune diseases can compromise BBB integrity, allowing peripheral immune cells and inflammatory mediators to infiltrate the central nervous system and potentially trigger migraine attacks. Such disruption exacerbates neuroinflammation, contributing to migraine pathogenesis. Accordingly, future research should elucidate the specific cellular and molecular changes associated with the comorbidity of autoimmune diseases and migraines. By understanding and targeting these biological mechanisms, the complex interplay between autoimmune diseases and migraines can be addressed, ultimately improving patient outcomes and quality of life.

Some recent studies on neuroimaging may shed some light. Increased brainstem activity is associated with pathways of pain and sensory processing and migraine (32–34). The hypothalamus is involved in regulation of homeostasis and is associated with migraine precursor symptoms (appetite, mood, sleep, etc.), suggesting that it may be involved in the regulation of migraine susceptibility (35). The genetic association observed in our study may be related to these neural mechanisms. The association of autoimmune hyperthyroidism with migraine may be related to brainstem and hypothalamic function, as endocrine regulation and pain processing are in these areas (34, 35). Cortical diffusive inhibition may be associated between SLE and MA, as seen on imaging (36, 37).

Thyroid hormones produced by the thyroid gland are particularly important for the growth of the neural system (38). Autoimmune hyperthyroidism and thyroid dysfunction often occur with migraine (39). A study in Denmark showed a notable increase in the proportion of migraine patients with hypothyroidism (*p* < 0.001, OR 95% CI = 2.11 [1.50–2.97]), and an increase in hyperthyroidism (p < 0.001, OR 95% CI = 1.80 [1.30–2.49]) (40). Migraine onset is more frequent in patients with a history of hypothyroidism (41). In an Italian case–control study, patients with subclinical hypothyroidism had a significantly higher prevalence of migraine (46%) than that of the control subjects (13%). Similarly, the study also found that individuals experiencing both subclinical hypothyroidism and migraine were more likely to develop other autoimmune diseases and at a higher risk than those with subclinical hypothyroidism alone (no migraine) (42). Elevated serum thyroid-stimulating hormone (TSH) levels indicate decreased thyroid function, whereas decreased TSH levels indicate hyperthyroidism. Previous studies found a notable correlation between migraine pathogenesis and fluctuations in TSH levels (43, 44). Given that the results of numerous studies showed discrepancies or contradictions in specific statistical results and relationships with direction, we conducted MR studies of both and found a causal and positive association between autoimmune hypersensitivity and migraine (migraine, MA, MO). Moreover, hypothyroidism was positively associated with MO but not with other migraine subtypes.

Calcitonin gene-related peptide (CGRP) levels have been reported to be upregulated in thyroid dysfunction (45). In addition, CGRP is known to play an important role during migraine and elevated levels can trigger migraine attacks (46). This suggests that increased CGRP levels in hypothyroidism/hyperthyroidism patients may increase susceptibility to migraine attacks. There are hypotheses that thyroid hormones may increase oxidative stress in the brain, causing vasospasm and vasoconstriction, which in turn triggers headaches (47). This may explain the association between hyperthyroidism and hypothyroidism and headache observed in this study. Therefore, clinical vigilance should be exercised, and patients with thyroid dysfunction should consider the possibility of subsequent migraines.

SLE has a wide range of symptoms, with neurological problems occurring in approximately 25%–75% of cases. Most patients with neurological involvement present with migraines (48). Individuals diagnosed with SLE are more likely to have headaches associated with cerebrovascular disease, and complications of SLE treatment may also cause migraine headaches (49–52). Some authors believe that the incidence of MA is high among SLE patients (53). This finding is consistent with the findings of the present study, suggesting a positive causal relationship between SLE and MA. MA may be related to the complex pathogenesis of neuropsychiatric manifestations of SLE. The factors involved were genetic factors, vascular damage and obstruction, blood–brain barrier dysfunction, direct neuronal self-injury, and death due to injury from inflammatory mediators or autoantibodies (54). Anti-β2-glycoprotein I antibodies (β2GPI) and anticardiolipin antibodies (aCL) levels were elevated in both SLE and patients with migraine (55–57). TNF-α, trigeminal ganglion and CGRP are involved in the pathogenesis of migraine with aura. This suggests that aCL and β2GPI may contribute to migraine with aura via TNF-α mediated pathways. In terms of vascular pathophysiology, antiphospholipid antibodies (aPL) and β2GPI activate endothelial cells and subsequently regulate serotonin and endothelin-1, which is involved in vasoconstriction and leads to migraine aura (58, 59). In general, genetic factors may lead to individual differences in SLE patients in various aspects such as immunology, vasculature, and neurology, thus increasing susceptibility to MA. This result highlights the importance of routine assessment and early identification of headache in SLE patients in clinical practice, which can help reduce unnecessary examinations and treatments.

Despite offering useful insights into the link between autoimmune diseases and migraines, this study has a few limitations. First, we acknowledge that the migraine diagnoses in the migraine dataset used in our study were primarily self-reported by the patient, introducing a probability of misdiagnosis. This limitation may affect the conclusions of the study. Self-reported data may sometimes lack accuracy for clinical diagnosis, as patients may misinterpret or inaccurately report their symptoms. Future studies should confirm these findings using clinically validated migraine diagnoses to improve data accuracy and validity. Second, we exclusively used exposure and outcome genomic data from European cohorts, restricting the generalizability of our findings to populations with high ethnic and genetic diversity. Therefore, the conclusions drawn from the study may be limited in relation to populations with high ethnic and genetic diversity. Additionally, future studies should consider diverse ethnic groups to validate these findings to different populations with high ethnic and genetic diversity. There are also cases in which sample overlap may bias the estimates from two-sample MR studies. The comorbidity of multiple autoimmune diseases may occur during the study, which may bias the results. Therefore, future research should consider examining the combined effects of multiple autoimmune diseases on migraine risk to provide a more comprehensive understanding. Third, there were limitations to the scope of this study, particularly in exploring the causal link between common autoimmune diseases and migraine, as not all patients with autoimmune disease were included in this study. While our study focused on 10 common autoimmune diseases, we did not consider all autoimmune conditions; therefore, limiting the exploration of potential causal links between other autoimmune diseases and migraines. As such, future research should consider examining a broad range of autoimmune disorders to provide a comprehensive understanding of the potential causal links. Fourth, the genetic link between autoimmune diseases and migraines remains unclear. Although we used the PhenoScanner GWAS dataset to evaluate the association of IV with confounding factors, it was unable to completely eliminate the influence of other factors, such as unmeasured genetic pleiotropy, population stratification, and environmental influences. Fifth, although we performed a comprehensive sensitivity analysis and the results showed no pleiotropy, there is a possibility of bias in our MR result, as complete exclusion of pleiotropy cannot be guaranteed. These confounding factors may include unmeasured genetic pleiotropy, population stratification, and environmental influences, which may bias these results. Future research should also conduct in-depth sensitivity analyses and use advanced statistical techniques to minimize potential biases. Future studies should therefore aim to incorporate more comprehensive datasets and methods to account for these potential confounders. This could include using finer genetic tools, larger and more diverse cohorts, and integrating environmental and lifestyle data to better understand the causal pathways involved. By addressing these limitations, future studies can enhance the robustness and applicability of the findings, ultimately improving our understanding of the relationship between autoimmune diseases and migraines.

## 5 Conclusion

A genetic correlation between autoimmune hyperthyroidism and migraine (migraine, MA, and MO) was confirmed in our study. Importantly, our study suggests that autoimmune hyperthyroidism may trigger migraine. In addition, SLE positively affects MA, and hypothyroidism contributes to the incidence of MO. The results of this study should be replicated and validated in other populations to ensure universality and robustness. At present, the outcome evidence does not suggest a significant causal connection between the selected immune disorders and migraine. This study reveals the potential interactions between some autoimmune diseases and migraine, providing valuable insights for future research and emphasizing the importance of considering these interactions in formulating public health policies and migraine prevention strategies. Future research directions are suggested to provide mechanistic insights into the causal relationship between autoimmune diseases and migraine, explore potential biological pathways and potential therapeutic targets.

## Data availability statement

The original contributions presented in the study are included in the article/Supplementary material, further inquiries can be directed to the corresponding authors.

## Ethics statement

Ethical review and approval was not required for the study on human participants in accordance with the local legislation and institutional requirements. Written informed consent from the patients/participants or patients/participants' legal guardian/next of kin was not required to participate in this study in accordance with the national legislation and the institutional requirements.

## Author contributions

RL: Writing – original draft. JH: Writing – review & editing. GS: Data curation, Writing – original draft. CL: Writing – original draft. SL: Writing – original draft. MW: Writing – original draft, Writing – review & editing. DY: Writing – original draft, Writing – review & editing.

## Footnotes

^1^ https://gwas.mrcieu.ac.uk/
