# Supplementary material for: Causality between multiple autoimmune disorders and migraine and its subtypes: a two-sample Mendelian randomization study
Source: Front Neurol. 2024 Jul 17;15:1420201. doi: 10.3389/fneur.2024.1420201 (PMC11288874; doi:10.3389/fneur.2024.1420201)
Supplement: Supplementary file 1 [file Table_1.DOCX]

Supplementary Material

Causality between multiple autoimmune disorders and migraine and its subtypes: A two-sample Mendelian randomization study

**Rui Li^1^, Jing Han^1,2^, Guoliang Shao^1^, Changyue Liu^1^, Shuo Li^1^, Mengmeng Wang^1,2,*^, and Dianhui Yang^1,2,*^**

***Correspondence:**

Mengmeng Wang: llliii2024@163.com

Dianhui Yang: szylrby@126.com

**Supplementary Tables**

**Supplementary Table 1 Detailed information of the studies and datasets used for Mendelian randomization analysis**

| Phenotypes | GWAS ID | Cases | Controls | Sample size | Population | Study/Consortium | PubMed ID | Year |
| --- | --- | --- | --- | --- | --- | --- | --- | --- |
|  |  |  |  |  |  |  |  |  |
| Celiac disease | ieu-a-1058 | 12041 | 12228 | 24269 | European | Trynka et al. | 22057235 | 2011 |
| Rheumatoid arthritis | ieu-a-833 | 19234 | 61565 | 80799 | European | Okada et al. | 24390342 | 2014 |
| Primary sclerosing cholangitis | ieu-a-1112 | 2871 | 12019 | 14890 | Mixed | IPSCSG | 27992413 | 2017 |
| Inflammatory bowel disease | ieu-a-294 | 31665 | 33977 | 65642 | European | IIBDGC | 26192919 | 2015 |
| Ulcerative colitis | ieu-a-32 | 6968 | 20464 | 27432 | European | IIBDGC | 26192919 | 2015 |
| Hypothyroidism | ebi-a-GCST90018862 | 30155 | 379986 | 410141 | European | Sakaue S et al. | 34594039 | 2021 |
| Autoimmune hyperthyroidism | finn-b-AUTOIMMUNE_HYPERTHYROIDISM | 962 | 172976 | 173938 | European | NA | - | 2021 |
| Type 1 diabetes | ebi-a-GCST90000529 | 7467 | 10218 | 17685 | European | Inshaw JRJ et al. | 33830302 | 2021 |
| Systemic lupus erythematosus | ebi-a-GCST003156 | 5201 | 9066 | 14267 | European | Bentham et al. | 26502338 | 2015 |
| Ankylosing spondylitis | ebi-a-GCST005529 | 9069 | 13578 | 22647 | European | IGAS | 23749187 | 2013 |
| Migraine | | | | | | | | |
| Migraine | finn-b-G6_MIGRAINE | 8547 | 176107 | 184654 | European | FinnGen | - | 2021 |
| Migraine with aura | finn-b-G6_MIGRAINE_WITH_AURA | 3541 | 176107 | 179648 | European | FinnGen | - | 2021 |
| Migraine without aura | finn-b-G6_MIGRAINE_NO_AURA | 3215 | 176107 | 179322 | European | FinnGen | - | 2021 |

**Supplementary Table 2 Analyzing the relationship between genetic prediction of autoimmune diseases and migraine by various MR analyses.**

| id.exposure | id.outcome | Outcome | Exposure | Method | nsnp | b | se | pval | or | or_lci95 | or_uci95 |
| --- | --- | --- | --- | --- | --- | --- | --- | --- | --- | --- | --- |
| ieu-a-1058 | finn-b-G6_MIGRAINE | Migraine \|\| id:finn-b-G6_MIGRAINE | Celiac disease \|\| id:ieu-a-1058 | MR Egger | 15 | -0.01626006 | 0.02132779 | 0.459 | 0.984 | 0.944 | 1.026 |
| ieu-a-1058 | finn-b-G6_MIGRAINE | Migraine \|\| id:finn-b-G6_MIGRAINE | Celiac disease \|\| id:ieu-a-1058 | Weighted median | 15 | 0.00307875 | 0.01723974 | 0.858 | 1.003 | 0.970 | 1.038 |
| ieu-a-1058 | finn-b-G6_MIGRAINE | Migraine \|\| id:finn-b-G6_MIGRAINE | Celiac disease \|\| id:ieu-a-1058 | Inverse variance weighted | 15 | 0.008367845 | 0.01516213 | 0.581 | 1.008 | 0.979 | 1.039 |
| ieu-a-1058 | finn-b-G6_MIGRAINE | Migraine \|\| id:finn-b-G6_MIGRAINE | Celiac disease \|\| id:ieu-a-1058 | Simple mode | 15 | 0.018828169 | 0.02637728 | 0.487 | 1.019 | 0.968 | 1.073 |
| ieu-a-1058 | finn-b-G6_MIGRAINE | Migraine \|\| id:finn-b-G6_MIGRAINE | Celiac disease \|\| id:ieu-a-1058 | Weighted mode | 15 | 0.00430071 | 0.01483401 | 0.776 | 1.004 | 0.976 | 1.034 |
| ieu-a-833 | finn-b-G6_MIGRAINE | Migraine \|\| id:finn-b-G6_MIGRAINE | Rheumatoid arthritis \|\| id:ieu-a-833 | MR Egger | 54 | -0.00541514 | 0.02823671 | 0.849 | 0.995 | 0.941 | 1.051 |
| ieu-a-833 | finn-b-G6_MIGRAINE | Migraine \|\| id:finn-b-G6_MIGRAINE | Rheumatoid arthritis \|\| id:ieu-a-833 | Weighted median | 54 | -0.01305834 | 0.02280128 | 0.567 | 0.987 | 0.944 | 1.032 |
| ieu-a-833 | finn-b-G6_MIGRAINE | Migraine \|\| id:finn-b-G6_MIGRAINE | Rheumatoid arthritis \|\| id:ieu-a-833 | Inverse variance weighted | 54 | 0.004207177 | 0.01798196 | 0.815 | 1.004 | 0.969 | 1.040 |
| ieu-a-833 | finn-b-G6_MIGRAINE | Migraine \|\| id:finn-b-G6_MIGRAINE | Rheumatoid arthritis \|\| id:ieu-a-833 | Simple mode | 54 | -0.01589821 | 0.048563 | 0.745 | 0.984 | 0.895 | 1.083 |
| ieu-a-833 | finn-b-G6_MIGRAINE | Migraine \|\| id:finn-b-G6_MIGRAINE | Rheumatoid arthritis \|\| id:ieu-a-833 | Weighted mode | 54 | -0.02399422 | 0.02150271 | 0.270 | 0.976 | 0.936 | 1.018 |
| ieu-a-1112 | finn-b-G6_MIGRAINE | Migraine \|\| id:finn-b-G6_MIGRAINE | Primary sclerosing cholangitis \|\| id:ieu-a-1112 | MR Egger | 18 | 0.03070408 | 0.03276544 | 0.363 | 1.031 | 0.967 | 1.100 |
| ieu-a-1112 | finn-b-G6_MIGRAINE | Migraine \|\| id:finn-b-G6_MIGRAINE | Primary sclerosing cholangitis \|\| id:ieu-a-1112 | Weighted median | 18 | 0.047078552 | 0.02344305 | 0.045 | 1.048 | 1.001 | 1.097 |
| ieu-a-1112 | finn-b-G6_MIGRAINE | Migraine \|\| id:finn-b-G6_MIGRAINE | Primary sclerosing cholangitis \|\| id:ieu-a-1112 | Inverse variance weighted | 18 | 0.022390926 | 0.0178263 | 0.209 | 1.023 | 0.988 | 1.059 |
| ieu-a-1112 | finn-b-G6_MIGRAINE | Migraine \|\| id:finn-b-G6_MIGRAINE | Primary sclerosing cholangitis \|\| id:ieu-a-1112 | Simple mode | 18 | -0.00712795 | 0.0405489 | 0.863 | 0.993 | 0.917 | 1.075 |
| ieu-a-1112 | finn-b-G6_MIGRAINE | Migraine \|\| id:finn-b-G6_MIGRAINE | Primary sclerosing cholangitis \|\| id:ieu-a-1112 | Weighted mode | 18 | 0.043112668 | 0.02404673 | 0.091 | 1.044 | 0.996 | 1.094 |
| ieu-a-294 | finn-b-G6_MIGRAINE | Migraine \|\| id:finn-b-G6_MIGRAINE | Inflammatory bowel disease \|\| id:ieu-a-294 | MR Egger | 132 | -0.0530276 | 0.037217 | 0.157 | 0.948 | 0.882 | 1.020 |
| ieu-a-294 | finn-b-G6_MIGRAINE | Migraine \|\| id:finn-b-G6_MIGRAINE | Inflammatory bowel disease \|\| id:ieu-a-294 | Weighted median | 132 | -0.00382732 | 0.02355582 | 0.871 | 0.996 | 0.951 | 1.043 |
| ieu-a-294 | finn-b-G6_MIGRAINE | Migraine \|\| id:finn-b-G6_MIGRAINE | Inflammatory bowel disease \|\| id:ieu-a-294 | Inverse variance weighted | 132 | -0.00354965 | 0.01538164 | 0.817 | 0.996 | 0.967 | 1.027 |
| ieu-a-294 | finn-b-G6_MIGRAINE | Migraine \|\| id:finn-b-G6_MIGRAINE | Inflammatory bowel disease \|\| id:ieu-a-294 | Simple mode | 132 | 0.022328842 | 0.04961959 | 0.653 | 1.023 | 0.928 | 1.127 |
| ieu-a-294 | finn-b-G6_MIGRAINE | Migraine \|\| id:finn-b-G6_MIGRAINE | Inflammatory bowel disease \|\| id:ieu-a-294 | Weighted mode | 132 | 0.012511931 | 0.03376971 | 0.712 | 1.013 | 0.948 | 1.082 |
| ieu-a-32 | finn-b-G6_MIGRAINE | Migraine \|\| id:finn-b-G6_MIGRAINE | Ulcerative colitis \|\| id:ieu-a-32 | MR Egger | 35 | 0.02682241 | 0.04931358 | 0.590 | 1.027 | 0.933 | 1.131 |
| ieu-a-32 | finn-b-G6_MIGRAINE | Migraine \|\| id:finn-b-G6_MIGRAINE | Ulcerative colitis \|\| id:ieu-a-32 | Weighted median | 35 | 0.04873444 | 0.0242472 | 0.044 | 1.050 | 1.001 | 1.101 |
| ieu-a-32 | finn-b-G6_MIGRAINE | Migraine \|\| id:finn-b-G6_MIGRAINE | Ulcerative colitis \|\| id:ieu-a-32 | Inverse variance weighted | 35 | 0.03223496 | 0.01698765 | 0.058 | 1.033 | 0.999 | 1.068 |
| ieu-a-32 | finn-b-G6_MIGRAINE | Migraine \|\| id:finn-b-G6_MIGRAINE | Ulcerative colitis \|\| id:ieu-a-32 | Simple mode | 35 | 0.02726206 | 0.05132621 | 0.599 | 1.028 | 0.929 | 1.136 |
| ieu-a-32 | finn-b-G6_MIGRAINE | Migraine \|\| id:finn-b-G6_MIGRAINE | Ulcerative colitis \|\| id:ieu-a-32 | Weighted mode | 35 | 0.04839402 | 0.03787478 | 0.210 | 1.050 | 0.974 | 1.130 |
| ebi-a-GCST90000529 | finn-b-G6_MIGRAINE | Migraine \|\| id:finn-b-G6_MIGRAINE | Type 1 diabetes \|\| id:ebi-a-GCST90000529 | MR Egger | 31 | 0.005930072 | 0.01179179 | 0.619 | 1.006 | 0.983 | 1.029 |
| ebi-a-GCST90000529 | finn-b-G6_MIGRAINE | Migraine \|\| id:finn-b-G6_MIGRAINE | Type 1 diabetes \|\| id:ebi-a-GCST90000529 | Weighted median | 31 | 0.008941878 | 0.009598643 | 0.352 | 1.009 | 0.990 | 1.028 |
| ebi-a-GCST90000529 | finn-b-G6_MIGRAINE | Migraine \|\| id:finn-b-G6_MIGRAINE | Type 1 diabetes \|\| id:ebi-a-GCST90000529 | Inverse variance weighted | 31 | -0.00556923 | 0.009174074 | 0.544 | 0.994 | 0.977 | 1.012 |
| ebi-a-GCST90000529 | finn-b-G6_MIGRAINE | Migraine \|\| id:finn-b-G6_MIGRAINE | Type 1 diabetes \|\| id:ebi-a-GCST90000529 | Simple mode | 31 | -0.00704688 | 0.031511174 | 0.825 | 0.993 | 0.934 | 1.056 |
| ebi-a-GCST90000529 | finn-b-G6_MIGRAINE | Migraine \|\| id:finn-b-G6_MIGRAINE | Type 1 diabetes \|\| id:ebi-a-GCST90000529 | Weighted mode | 31 | 0.006753559 | 0.008934843 | 0.456 | 1.007 | 0.989 | 1.025 |
| ebi-a-GCST90018862 | finn-b-G6_MIGRAINE | Migraine \|\| id:finn-b-G6_MIGRAINE | Hypothyroidism \|\| id:ebi-a-GCST90018862 | MR Egger | 68 | -0.00741558 | 0.04791657 | 0.877 | 0.993 | 0.904 | 1.090 |
| ebi-a-GCST90018862 | finn-b-G6_MIGRAINE | Migraine \|\| id:finn-b-G6_MIGRAINE | Hypothyroidism \|\| id:ebi-a-GCST90018862 | Weighted median | 68 | 0.032206818 | 0.03363963 | 0.338 | 1.033 | 0.967 | 1.103 |
| ebi-a-GCST90018862 | finn-b-G6_MIGRAINE | Migraine \|\| id:finn-b-G6_MIGRAINE | Hypothyroidism \|\| id:ebi-a-GCST90018862 | Inverse variance weighted | 68 | 0.043675673 | 0.02236132 | 0.051 | 1.045 | 1.000 | 1.091 |
| ebi-a-GCST90018862 | finn-b-G6_MIGRAINE | Migraine \|\| id:finn-b-G6_MIGRAINE | Hypothyroidism \|\| id:ebi-a-GCST90018862 | Simple mode | 68 | 0.081720998 | 0.06537779 | 0.216 | 1.085 | 0.955 | 1.234 |
| ebi-a-GCST90018862 | finn-b-G6_MIGRAINE | Migraine \|\| id:finn-b-G6_MIGRAINE | Hypothyroidism \|\| id:ebi-a-GCST90018862 | Weighted mode | 68 | 0.014193985 | 0.04195412 | 0.736 | 1.014 | 0.934 | 1.101 |
| finn-b-AUTOIMMUNE_HYPERTHYROIDISM | finn-b-G6_MIGRAINE | Migraine \|\| id:finn-b-G6_MIGRAINE | Autoimmune hyperthyroidism \|\| id:finn-b-AUTOIMMUNE_HYPERTHYROIDISM | MR Egger | 7 | 0.0587584 | 0.04252447 | 0.226 | 1.061 | 0.976 | 1.153 |
| finn-b-AUTOIMMUNE_HYPERTHYROIDISM | finn-b-G6_MIGRAINE | Migraine \|\| id:finn-b-G6_MIGRAINE | Autoimmune hyperthyroidism \|\| id:finn-b-AUTOIMMUNE_HYPERTHYROIDISM | Weighted median | 7 | 0.06304673 | 0.02236989 | 0.005 | 1.065 | 1.019 | 1.113 |
| finn-b-AUTOIMMUNE_HYPERTHYROIDISM | finn-b-G6_MIGRAINE | Migraine \|\| id:finn-b-G6_MIGRAINE | Autoimmune hyperthyroidism \|\| id:finn-b-AUTOIMMUNE_HYPERTHYROIDISM | Inverse variance weighted | 7 | 0.06367793 | 0.01752241 | 0.000 | 1.066 | 1.030 | 1.103 |
| finn-b-AUTOIMMUNE_HYPERTHYROIDISM | finn-b-G6_MIGRAINE | Migraine \|\| id:finn-b-G6_MIGRAINE | Autoimmune hyperthyroidism \|\| id:finn-b-AUTOIMMUNE_HYPERTHYROIDISM | Simple mode | 7 | 0.06446073 | 0.03080022 | 0.081 | 1.067 | 1.004 | 1.133 |
| finn-b-AUTOIMMUNE_HYPERTHYROIDISM | finn-b-G6_MIGRAINE | Migraine \|\| id:finn-b-G6_MIGRAINE | Autoimmune hyperthyroidism \|\| id:finn-b-AUTOIMMUNE_HYPERTHYROIDISM | Weighted mode | 7 | 0.05863108 | 0.02740254 | 0.076 | 1.060 | 1.005 | 1.119 |
| ebi-a-GCST003156 | finn-b-G6_MIGRAINE | Migraine \|\| id:finn-b-G6_MIGRAINE | Systemic lupus erythematosus \|\| id:ebi-a-GCST003156 | MR Egger | 42 | 0.034767962 | 0.02387162 | 0.153 | 1.035 | 0.988 | 1.085 |
| ebi-a-GCST003156 | finn-b-G6_MIGRAINE | Migraine \|\| id:finn-b-G6_MIGRAINE | Systemic lupus erythematosus \|\| id:ebi-a-GCST003156 | Weighted median | 42 | -0.00153469 | 0.01438239 | 0.915 | 0.998 | 0.971 | 1.027 |
| ebi-a-GCST003156 | finn-b-G6_MIGRAINE | Migraine \|\| id:finn-b-G6_MIGRAINE | Systemic lupus erythematosus \|\| id:ebi-a-GCST003156 | Inverse variance weighted | 42 | 0.012075052 | 0.01103785 | 0.274 | 1.012 | 0.990 | 1.034 |
| ebi-a-GCST003156 | finn-b-G6_MIGRAINE | Migraine \|\| id:finn-b-G6_MIGRAINE | Systemic lupus erythematosus \|\| id:ebi-a-GCST003156 | Simple mode | 42 | -0.00068187 | 0.02798787 | 0.981 | 0.999 | 0.946 | 1.056 |
| ebi-a-GCST003156 | finn-b-G6_MIGRAINE | Migraine \|\| id:finn-b-G6_MIGRAINE | Systemic lupus erythematosus \|\| id:ebi-a-GCST003156 | Weighted mode | 42 | -0.01340502 | 0.02208576 | 0.547 | 0.987 | 0.945 | 1.030 |
| ebi-a-GCST005529 | finn-b-G6_MIGRAINE | Migraine \|\| id:finn-b-G6_MIGRAINE | Ankylosing spondylitis \|\| id:ebi-a-GCST005529 | MR Egger | 24 | -0.15999076 | 0.12998865 | 0.231 | 0.852 | 0.660 | 1.099 |
| ebi-a-GCST005529 | finn-b-G6_MIGRAINE | Migraine \|\| id:finn-b-G6_MIGRAINE | Ankylosing spondylitis \|\| id:ebi-a-GCST005529 | Weighted median | 24 | -0.09268283 | 0.11139209 | 0.405 | 0.911 | 0.733 | 1.134 |
| ebi-a-GCST005529 | finn-b-G6_MIGRAINE | Migraine \|\| id:finn-b-G6_MIGRAINE | Ankylosing spondylitis \|\| id:ebi-a-GCST005529 | Inverse variance weighted | 24 | -0.1273956 | 0.07521855 | 0.090 | 0.880 | 0.760 | 1.020 |
| ebi-a-GCST005529 | finn-b-G6_MIGRAINE | Migraine \|\| id:finn-b-G6_MIGRAINE | Ankylosing spondylitis \|\| id:ebi-a-GCST005529 | Simple mode | 24 | -0.0267605 | 0.20411154 | 0.897 | 0.974 | 0.653 | 1.453 |
| ebi-a-GCST005529 | finn-b-G6_MIGRAINE | Migraine \|\| id:finn-b-G6_MIGRAINE | Ankylosing spondylitis \|\| id:ebi-a-GCST005529 | Weighted mode | 24 | -0.10116696 | 0.10380665 | 0.340 | 0.904 | 0.737 | 1.108 |

**Supplementary Table 3 Heterogeneity and pleiotropy tests**

| Exposure | Outcome | Heterogeneity test | | | | | | Pleiotropy test | | |
| --- | --- | --- | --- | --- | --- | --- | --- | --- | --- | --- |
|  |  | MR Egger | | | Inverse variance weighted | | | MR Egger | | |
|  |  | Q | Q_df | p-value | Q | Q_df | p-value | Intercept | SE | p-value |
| Primary sclerosing cholangitis | Migraine | 20.7651 | 16 | 0.1877176 | 20.88665 | 17 | 0.2314017 | -0.00357691 | 0.01168803 | 0.7635264 |
| Ulcerative colitis | Migraine | 30.26111 | 33 | 0.6041809 | 30.27478 | 34 | 0.6508714 | 0.001080228 | 0.009239544 | 0.9076372 |
| Ulcerative colitis | MO | 33.49364 | 33 | 0.4433272 | 35.819 | 34 | 0.383090 | -0.02213568 | 0.01462419 | 0.1396382 |
| Hypothyroidism | MO | 65.60871 | 66 | 0.4904379 | 66.27515 | 67 | 0.5020587 | 0.006400164 | 0.007839859 | 0.4172299 |
| Autoimmune hyperthyroidism | Migraine | 5.307858 | 5 | 0.379475 | 5.325189 | 6 | 0.5028315 | 0.002906716 | 0.02274889 | 0.9033078 |
| Autoimmune hyperthyroidism | MA | 0.5060512 | 5 | 0.9919 | 0.6529866 | 6 | 0.99545 | -0.01266748 | 0.03304662 | 0.7172381 |
| Autoimmune hyperthyroidism | MO | 4.619394 | 5 | 0.4640695 | 4.691416 | 6 | 0.5839554 | 0.009317234 | 0.03471799 | 0.7991356 |
| Systemic lupus erythematosus | MA | 39.81167 | 40 | 0.4786415 | 40.41548 | 41 | 0.4964356 | -0.008628074 | 0.01110357 | 0.4416984 |

MR, Mendelian randomization; Q, heterogeneity statistic Q; df, degree of freedom; SE, standard error

**Supplementary Table 4 Analyzing the relationship between genetic prediction of autoimmune diseases and migraine subtypes by various MR analyses.**

| id.exposure | id.outcome | outcome | exposure | method | nsnp | b | se | pval | or | or_lci95 | or_uci95 |
| --- | --- | --- | --- | --- | --- | --- | --- | --- | --- | --- | --- |
| ieu-a-1058 | finn-b-G6_MIGRAINE_WITH_AURA | Migraine with aura \|\| id:finn-b-G6_MIGRAINE_WITH_AURA | Celiac disease \|\| id:ieu-a-1058 | MR Egger | 15 | -0.01459 | 0.031054 | 0.646 | 0.986 | 0.927 | 1.047 |
| ieu-a-1058 | finn-b-G6_MIGRAINE_WITH_AURA | Migraine with aura \|\| id:finn-b-G6_MIGRAINE_WITH_AURA | Celiac disease \|\| id:ieu-a-1058 | Weighted median | 15 | -0.00046 | 0.027676 | 0.987 | 1.000 | 0.947 | 1.055 |
| ieu-a-1058 | finn-b-G6_MIGRAINE_WITH_AURA | Migraine with aura \|\| id:finn-b-G6_MIGRAINE_WITH_AURA | Celiac disease \|\| id:ieu-a-1058 | Inverse variance weighted | 15 | 0.013516 | 0.021392 | 0.527 | 1.014 | 0.972 | 1.057 |
| ieu-a-1058 | finn-b-G6_MIGRAINE_WITH_AURA | Migraine with aura \|\| id:finn-b-G6_MIGRAINE_WITH_AURA | Celiac disease \|\| id:ieu-a-1058 | Simple mode | 15 | 0.019881 | 0.038306 | 0.612 | 1.020 | 0.946 | 1.100 |
| ieu-a-1058 | finn-b-G6_MIGRAINE_WITH_AURA | Migraine with aura \|\| id:finn-b-G6_MIGRAINE_WITH_AURA | Celiac disease \|\| id:ieu-a-1058 | Weighted mode | 15 | 0.00146 | 0.024753 | 0.954 | 1.001 | 0.954 | 1.051 |
| ieu-a-1058 | finn-b-G6_MIGRAINE_NO_AURA | Migraine without aura \|\| id:finn-b-G6_MIGRAINE_NO_AURA | Celiac disease \|\| id:ieu-a-1058 | MR Egger | 15 | -0.03956 | 0.03156 | 0.232 | 0.961 | 0.904 | 1.023 |
| ieu-a-1058 | finn-b-G6_MIGRAINE_NO_AURA | Migraine without aura \|\| id:finn-b-G6_MIGRAINE_NO_AURA | Celiac disease \|\| id:ieu-a-1058 | Weighted median | 15 | -0.01926 | 0.027442 | 0.483 | 0.981 | 0.930 | 1.035 |
| ieu-a-1058 | finn-b-G6_MIGRAINE_NO_AURA | Migraine without aura \|\| id:finn-b-G6_MIGRAINE_NO_AURA | Celiac disease \|\| id:ieu-a-1058 | Inverse variance weighted | 15 | -0.02327 | 0.021347 | 0.276 | 0.977 | 0.937 | 1.019 |
| ieu-a-1058 | finn-b-G6_MIGRAINE_NO_AURA | Migraine without aura \|\| id:finn-b-G6_MIGRAINE_NO_AURA | Celiac disease \|\| id:ieu-a-1058 | Simple mode | 15 | -0.04386 | 0.040324 | 0.295 | 0.957 | 0.884 | 1.036 |
| ieu-a-1058 | finn-b-G6_MIGRAINE_NO_AURA | Migraine without aura \|\| id:finn-b-G6_MIGRAINE_NO_AURA | Celiac disease \|\| id:ieu-a-1058 | Weighted mode | 15 | -0.01696 | 0.025554 | 0.518 | 0.983 | 0.935 | 1.034 |
| ieu-a-833 | finn-b-G6_MIGRAINE_WITH_AURA | Migraine with aura \|\| id:finn-b-G6_MIGRAINE_WITH_AURA | Rheumatoid arthritis \|\| id:ieu-a-833 | MR Egger | 54 | 0.034931 | 0.036097 | 0.338 | 1.036 | 0.965 | 1.111 |
| ieu-a-833 | finn-b-G6_MIGRAINE_WITH_AURA | Migraine with aura \|\| id:finn-b-G6_MIGRAINE_WITH_AURA | Rheumatoid arthritis \|\| id:ieu-a-833 | Weighted median | 54 | -0.00873 | 0.033671 | 0.795 | 0.991 | 0.928 | 1.059 |
| ieu-a-833 | finn-b-G6_MIGRAINE_WITH_AURA | Migraine with aura \|\| id:finn-b-G6_MIGRAINE_WITH_AURA | Rheumatoid arthritis \|\| id:ieu-a-833 | Inverse variance weighted | 54 | 0.000831 | 0.023273 | 0.972 | 1.001 | 0.956 | 1.048 |
| ieu-a-833 | finn-b-G6_MIGRAINE_WITH_AURA | Migraine with aura \|\| id:finn-b-G6_MIGRAINE_WITH_AURA | Rheumatoid arthritis \|\| id:ieu-a-833 | Simple mode | 54 | 0.079429 | 0.070581 | 0.266 | 1.083 | 0.943 | 1.243 |
| ieu-a-833 | finn-b-G6_MIGRAINE_WITH_AURA | Migraine with aura \|\| id:finn-b-G6_MIGRAINE_WITH_AURA | Rheumatoid arthritis \|\| id:ieu-a-833 | Weighted mode | 54 | -0.00013 | 0.033515 | 0.997 | 1.000 | 0.936 | 1.068 |
| ieu-a-833 | finn-b-G6_MIGRAINE_NO_AURA | Migraine without aura \|\| id:finn-b-G6_MIGRAINE_NO_AURA | Rheumatoid arthritis \|\| id:ieu-a-833 | MR Egger | 54 | -0.03062 | 0.039921 | 0.447 | 0.970 | 0.897 | 1.049 |
| ieu-a-833 | finn-b-G6_MIGRAINE_NO_AURA | Migraine without aura \|\| id:finn-b-G6_MIGRAINE_NO_AURA | Rheumatoid arthritis \|\| id:ieu-a-833 | Weighted median | 54 | 0.003766 | 0.0396 | 0.924 | 1.004 | 0.929 | 1.085 |
| ieu-a-833 | finn-b-G6_MIGRAINE_NO_AURA | Migraine without aura \|\| id:finn-b-G6_MIGRAINE_NO_AURA | Rheumatoid arthritis \|\| id:ieu-a-833 | Inverse variance weighted | 54 | 0.007893 | 0.025748 | 0.759 | 1.008 | 0.958 | 1.060 |
| ieu-a-833 | finn-b-G6_MIGRAINE_NO_AURA | Migraine without aura \|\| id:finn-b-G6_MIGRAINE_NO_AURA | Rheumatoid arthritis \|\| id:ieu-a-833 | Simple mode | 54 | -0.07422 | 0.077622 | 0.343 | 0.928 | 0.797 | 1.081 |
| ieu-a-833 | finn-b-G6_MIGRAINE_NO_AURA | Migraine without aura \|\| id:finn-b-G6_MIGRAINE_NO_AURA | Rheumatoid arthritis \|\| id:ieu-a-833 | Weighted mode | 54 | -0.03238 | 0.035227 | 0.362 | 0.968 | 0.904 | 1.037 |
| ieu-a-1112 | finn-b-G6_MIGRAINE_WITH_AURA | Migraine with aura \|\| id:finn-b-G6_MIGRAINE_WITH_AURA | Primary sclerosing cholangitis \|\| id:ieu-a-1112 | MR Egger | 18 | 0.021719 | 0.055773 | 0.702 | 1.022 | 0.916 | 1.140 |
| ieu-a-1112 | finn-b-G6_MIGRAINE_WITH_AURA | Migraine with aura \|\| id:finn-b-G6_MIGRAINE_WITH_AURA | Primary sclerosing cholangitis \|\| id:ieu-a-1112 | Weighted median | 18 | 0.00732 | 0.035466 | 0.836 | 1.007 | 0.940 | 1.080 |
| ieu-a-1112 | finn-b-G6_MIGRAINE_WITH_AURA | Migraine with aura \|\| id:finn-b-G6_MIGRAINE_WITH_AURA | Primary sclerosing cholangitis \|\| id:ieu-a-1112 | Inverse variance weighted | 18 | 0.032179 | 0.030214 | 0.287 | 1.033 | 0.973 | 1.096 |
| ieu-a-1112 | finn-b-G6_MIGRAINE_WITH_AURA | Migraine with aura \|\| id:finn-b-G6_MIGRAINE_WITH_AURA | Primary sclerosing cholangitis \|\| id:ieu-a-1112 | Simple mode | 18 | 0.001554 | 0.063393 | 0.981 | 1.002 | 0.885 | 1.134 |
| ieu-a-1112 | finn-b-G6_MIGRAINE_WITH_AURA | Migraine with aura \|\| id:finn-b-G6_MIGRAINE_WITH_AURA | Primary sclerosing cholangitis \|\| id:ieu-a-1112 | Weighted mode | 18 | 0.025529 | 0.035829 | 0.486 | 1.026 | 0.956 | 1.100 |
| ieu-a-1112 | finn-b-G6_MIGRAINE_NO_AURA | Migraine without aura \|\| id:finn-b-G6_MIGRAINE_NO_AURA | Primary sclerosing cholangitis \|\| id:ieu-a-1112 | MR Egger | 18 | 0.010957 | 0.054377 | 0.843 | 1.011 | 0.909 | 1.125 |
| ieu-a-1112 | finn-b-G6_MIGRAINE_NO_AURA | Migraine without aura \|\| id:finn-b-G6_MIGRAINE_NO_AURA | Primary sclerosing cholangitis \|\| id:ieu-a-1112 | Weighted median | 18 | 0.027374 | 0.036078 | 0.448 | 1.028 | 0.958 | 1.103 |
| ieu-a-1112 | finn-b-G6_MIGRAINE_NO_AURA | Migraine without aura \|\| id:finn-b-G6_MIGRAINE_NO_AURA | Primary sclerosing cholangitis \|\| id:ieu-a-1112 | Inverse variance weighted | 18 | 0.016425 | 0.029502 | 0.578 | 1.017 | 0.959 | 1.077 |
| ieu-a-1112 | finn-b-G6_MIGRAINE_NO_AURA | Migraine without aura \|\| id:finn-b-G6_MIGRAINE_NO_AURA | Primary sclerosing cholangitis \|\| id:ieu-a-1112 | Simple mode | 18 | 0.045756 | 0.067026 | 0.504 | 1.047 | 0.918 | 1.194 |
| ieu-a-1112 | finn-b-G6_MIGRAINE_NO_AURA | Migraine without aura \|\| id:finn-b-G6_MIGRAINE_NO_AURA | Primary sclerosing cholangitis \|\| id:ieu-a-1112 | Weighted mode | 18 | 0.050075 | 0.038567 | 0.211 | 1.051 | 0.975 | 1.134 |
| ieu-a-294 | finn-b-G6_MIGRAINE_WITH_AURA | Migraine with aura \|\| id:finn-b-G6_MIGRAINE_WITH_AURA | Inflammatory bowel disease \|\| id:ieu-a-294 | MR Egger | 132 | -0.15758 | 0.05783 | 0.007 | 0.854 | 0.763 | 0.957 |
| ieu-a-294 | finn-b-G6_MIGRAINE_WITH_AURA | Migraine with aura \|\| id:finn-b-G6_MIGRAINE_WITH_AURA | Inflammatory bowel disease \|\| id:ieu-a-294 | Weighted median | 132 | -0.05497 | 0.035079 | 0.117 | 0.947 | 0.884 | 1.014 |
| ieu-a-294 | finn-b-G6_MIGRAINE_WITH_AURA | Migraine with aura \|\| id:finn-b-G6_MIGRAINE_WITH_AURA | Inflammatory bowel disease \|\| id:ieu-a-294 | Inverse variance weighted | 132 | -0.02263 | 0.024355 | 0.353 | 0.978 | 0.932 | 1.025 |
| ieu-a-294 | finn-b-G6_MIGRAINE_WITH_AURA | Migraine with aura \|\| id:finn-b-G6_MIGRAINE_WITH_AURA | Inflammatory bowel disease \|\| id:ieu-a-294 | Simple mode | 132 | -0.08996 | 0.080596 | 0.266 | 0.914 | 0.780 | 1.070 |
| ieu-a-294 | finn-b-G6_MIGRAINE_WITH_AURA | Migraine with aura \|\| id:finn-b-G6_MIGRAINE_WITH_AURA | Inflammatory bowel disease \|\| id:ieu-a-294 | Weighted mode | 132 | -0.08466 | 0.055447 | 0.129 | 0.919 | 0.824 | 1.024 |
| ieu-a-294 | finn-b-G6_MIGRAINE_NO_AURA | Migraine without aura \|\| id:finn-b-G6_MIGRAINE_NO_AURA | Inflammatory bowel disease \|\| id:ieu-a-294 | MR Egger | 132 | -0.00363 | 0.060075 | 0.952 | 0.996 | 0.886 | 1.121 |
| ieu-a-294 | finn-b-G6_MIGRAINE_NO_AURA | Migraine without aura \|\| id:finn-b-G6_MIGRAINE_NO_AURA | Inflammatory bowel disease \|\| id:ieu-a-294 | Weighted median | 132 | -0.01889 | 0.03658 | 0.605 | 0.981 | 0.913 | 1.054 |
| ieu-a-294 | finn-b-G6_MIGRAINE_NO_AURA | Migraine without aura \|\| id:finn-b-G6_MIGRAINE_NO_AURA | Inflammatory bowel disease \|\| id:ieu-a-294 | Inverse variance weighted | 132 | -0.00155 | 0.02465 | 0.950 | 0.998 | 0.951 | 1.048 |
| ieu-a-294 | finn-b-G6_MIGRAINE_NO_AURA | Migraine without aura \|\| id:finn-b-G6_MIGRAINE_NO_AURA | Inflammatory bowel disease \|\| id:ieu-a-294 | Simple mode | 132 | -0.05205 | 0.103585 | 0.616 | 0.949 | 0.775 | 1.163 |
| ieu-a-294 | finn-b-G6_MIGRAINE_NO_AURA | Migraine without aura \|\| id:finn-b-G6_MIGRAINE_NO_AURA | Inflammatory bowel disease \|\| id:ieu-a-294 | Weighted mode | 132 | -0.03662 | 0.072102 | 0.612 | 0.964 | 0.837 | 1.110 |
| ieu-a-32 | finn-b-G6_MIGRAINE_WITH_AURA | Migraine with aura \|\| id:finn-b-G6_MIGRAINE_WITH_AURA | Ulcerative colitis \|\| id:ieu-a-32 | MR Egger | 35 | -0.04803 | 0.074228 | 0.522 | 0.953 | 0.824 | 1.102 |
| ieu-a-32 | finn-b-G6_MIGRAINE_WITH_AURA | Migraine with aura \|\| id:finn-b-G6_MIGRAINE_WITH_AURA | Ulcerative colitis \|\| id:ieu-a-32 | Weighted median | 35 | 0.023223 | 0.038203 | 0.543 | 1.023 | 0.950 | 1.103 |
| ieu-a-32 | finn-b-G6_MIGRAINE_WITH_AURA | Migraine with aura \|\| id:finn-b-G6_MIGRAINE_WITH_AURA | Ulcerative colitis \|\| id:ieu-a-32 | Inverse variance weighted | 35 | 0.027901 | 0.025676 | 0.277 | 1.028 | 0.978 | 1.081 |
| ieu-a-32 | finn-b-G6_MIGRAINE_WITH_AURA | Migraine with aura \|\| id:finn-b-G6_MIGRAINE_WITH_AURA | Ulcerative colitis \|\| id:ieu-a-32 | Simple mode | 35 | -0.01939 | 0.081414 | 0.813 | 0.981 | 0.836 | 1.150 |
| ieu-a-32 | finn-b-G6_MIGRAINE_WITH_AURA | Migraine with aura \|\| id:finn-b-G6_MIGRAINE_WITH_AURA | Ulcerative colitis \|\| id:ieu-a-32 | Weighted mode | 35 | -0.0026 | 0.066272 | 0.969 | 0.997 | 0.876 | 1.136 |
| ieu-a-32 | finn-b-G6_MIGRAINE_NO_AURA | Migraine without aura \|\| id:finn-b-G6_MIGRAINE_NO_AURA | Ulcerative colitis \|\| id:ieu-a-32 | MR Egger | 35 | 0.142124 | 0.078035 | 0.078 | 1.153 | 0.989 | 1.343 |
| ieu-a-32 | finn-b-G6_MIGRAINE_NO_AURA | Migraine without aura \|\| id:finn-b-G6_MIGRAINE_NO_AURA | Ulcerative colitis \|\| id:ieu-a-32 | Weighted median | 35 | 0.084527 | 0.040179 | 0.035 | 1.088 | 1.006 | 1.177 |
| ieu-a-32 | finn-b-G6_MIGRAINE_NO_AURA | Migraine without aura \|\| id:finn-b-G6_MIGRAINE_NO_AURA | Ulcerative colitis \|\| id:ieu-a-32 | Inverse variance weighted | 35 | 0.031235 | 0.027382 | 0.254 | 1.032 | 0.978 | 1.089 |
| ieu-a-32 | finn-b-G6_MIGRAINE_NO_AURA | Migraine without aura \|\| id:finn-b-G6_MIGRAINE_NO_AURA | Ulcerative colitis \|\| id:ieu-a-32 | Simple mode | 35 | 0.122822 | 0.092077 | 0.191 | 1.131 | 0.944 | 1.354 |
| ieu-a-32 | finn-b-G6_MIGRAINE_NO_AURA | Migraine without aura \|\| id:finn-b-G6_MIGRAINE_NO_AURA | Ulcerative colitis \|\| id:ieu-a-32 | Weighted mode | 35 | 0.118229 | 0.056597 | 0.044 | 1.126 | 1.007 | 1.258 |
| ebi-a-GCST90000529 | finn-b-G6_MIGRAINE_WITH_AURA | Migraine with aura \|\| id:finn-b-G6_MIGRAINE_WITH_AURA | Type 1 diabetes \|\| id:ebi-a-GCST90000529 | MR Egger | 31 | 0.015616 | 0.014566 | 0.293 | 1.016 | 0.987 | 1.045 |
| ebi-a-GCST90000529 | finn-b-G6_MIGRAINE_WITH_AURA | Migraine with aura \|\| id:finn-b-G6_MIGRAINE_WITH_AURA | Type 1 diabetes \|\| id:ebi-a-GCST90000529 | Weighted median | 31 | 0.011222 | 0.014472 | 0.438 | 1.011 | 0.983 | 1.040 |
| ebi-a-GCST90000529 | finn-b-G6_MIGRAINE_WITH_AURA | Migraine with aura \|\| id:finn-b-G6_MIGRAINE_WITH_AURA | Type 1 diabetes \|\| id:ebi-a-GCST90000529 | Inverse variance weighted | 31 | -0.00087 | 0.011335 | 0.939 | 0.999 | 0.977 | 1.022 |
| ebi-a-GCST90000529 | finn-b-G6_MIGRAINE_WITH_AURA | Migraine with aura \|\| id:finn-b-G6_MIGRAINE_WITH_AURA | Type 1 diabetes \|\| id:ebi-a-GCST90000529 | Simple mode | 31 | -0.03091 | 0.040471 | 0.451 | 0.970 | 0.896 | 1.050 |
| ebi-a-GCST90000529 | finn-b-G6_MIGRAINE_WITH_AURA | Migraine with aura \|\| id:finn-b-G6_MIGRAINE_WITH_AURA | Type 1 diabetes \|\| id:ebi-a-GCST90000529 | Weighted mode | 31 | 0.008925 | 0.01284 | 0.492 | 1.009 | 0.984 | 1.035 |
| ebi-a-GCST90000529 | finn-b-G6_MIGRAINE_NO_AURA | Migraine without aura \|\| id:finn-b-G6_MIGRAINE_NO_AURA | Type 1 diabetes \|\| id:ebi-a-GCST90000529 | MR Egger | 31 | -0.00722 | 0.018532 | 0.700 | 0.993 | 0.957 | 1.030 |
| ebi-a-GCST90000529 | finn-b-G6_MIGRAINE_NO_AURA | Migraine without aura \|\| id:finn-b-G6_MIGRAINE_NO_AURA | Type 1 diabetes \|\| id:ebi-a-GCST90000529 | Weighted median | 31 | 0.01249 | 0.017246 | 0.469 | 1.013 | 0.979 | 1.047 |
| ebi-a-GCST90000529 | finn-b-G6_MIGRAINE_NO_AURA | Migraine without aura \|\| id:finn-b-G6_MIGRAINE_NO_AURA | Type 1 diabetes \|\| id:ebi-a-GCST90000529 | Inverse variance weighted | 31 | -0.01452 | 0.013978 | 0.299 | 0.986 | 0.959 | 1.013 |
| ebi-a-GCST90000529 | finn-b-G6_MIGRAINE_NO_AURA | Migraine without aura \|\| id:finn-b-G6_MIGRAINE_NO_AURA | Type 1 diabetes \|\| id:ebi-a-GCST90000529 | Simple mode | 31 | -0.0361 | 0.046164 | 0.440 | 0.965 | 0.881 | 1.056 |
| ebi-a-GCST90000529 | finn-b-G6_MIGRAINE_NO_AURA | Migraine without aura \|\| id:finn-b-G6_MIGRAINE_NO_AURA | Type 1 diabetes \|\| id:ebi-a-GCST90000529 | Weighted mode | 31 | -0.00391 | 0.014243 | 0.785 | 0.996 | 0.969 | 1.024 |
| ebi-a-GCST90018862 | finn-b-G6_MIGRAINE_WITH_AURA | Migraine with aura \|\| id:finn-b-G6_MIGRAINE_WITH_AURA | Hypothyroidism \|\| id:ebi-a-GCST90018862 | MR Egger | 68 | 0.051435 | 0.071549 | 0.475 | 1.053 | 0.915 | 1.211 |
| ebi-a-GCST90018862 | finn-b-G6_MIGRAINE_WITH_AURA | Migraine with aura \|\| id:finn-b-G6_MIGRAINE_WITH_AURA | Hypothyroidism \|\| id:ebi-a-GCST90018862 | Weighted median | 68 | 0.055617 | 0.054012 | 0.303 | 1.057 | 0.951 | 1.175 |
| ebi-a-GCST90018862 | finn-b-G6_MIGRAINE_WITH_AURA | Migraine with aura \|\| id:finn-b-G6_MIGRAINE_WITH_AURA | Hypothyroidism \|\| id:ebi-a-GCST90018862 | Inverse variance weighted | 68 | 0.040049 | 0.033354 | 0.230 | 1.041 | 0.975 | 1.111 |
| ebi-a-GCST90018862 | finn-b-G6_MIGRAINE_WITH_AURA | Migraine with aura \|\| id:finn-b-G6_MIGRAINE_WITH_AURA | Hypothyroidism \|\| id:ebi-a-GCST90018862 | Simple mode | 68 | 0.146211 | 0.098355 | 0.142 | 1.157 | 0.955 | 1.404 |
| ebi-a-GCST90018862 | finn-b-G6_MIGRAINE_WITH_AURA | Migraine with aura \|\| id:finn-b-G6_MIGRAINE_WITH_AURA | Hypothyroidism \|\| id:ebi-a-GCST90018862 | Weighted mode | 68 | 0.054644 | 0.062906 | 0.388 | 1.056 | 0.934 | 1.195 |
| ebi-a-GCST90018862 | finn-b-G6_MIGRAINE_NO_AURA | Migraine without aura \|\| id:finn-b-G6_MIGRAINE_NO_AURA | Hypothyroidism \|\| id:ebi-a-GCST90018862 | MR Egger | 68 | 0.01862 | 0.075261 | 0.805 | 1.019 | 0.879 | 1.181 |
| ebi-a-GCST90018862 | finn-b-G6_MIGRAINE_NO_AURA | Migraine without aura \|\| id:finn-b-G6_MIGRAINE_NO_AURA | Hypothyroidism \|\| id:ebi-a-GCST90018862 | Weighted median | 68 | 0.017704 | 0.053431 | 0.740 | 1.018 | 0.917 | 1.130 |
| ebi-a-GCST90018862 | finn-b-G6_MIGRAINE_NO_AURA | Migraine without aura \|\| id:finn-b-G6_MIGRAINE_NO_AURA | Hypothyroidism \|\| id:ebi-a-GCST90018862 | Inverse variance weighted | 68 | 0.072955 | 0.035133 | 0.038 | 1.076 | 1.004 | 1.152 |
| ebi-a-GCST90018862 | finn-b-G6_MIGRAINE_NO_AURA | Migraine without aura \|\| id:finn-b-G6_MIGRAINE_NO_AURA | Hypothyroidism \|\| id:ebi-a-GCST90018862 | Simple mode | 68 | 0.018319 | 0.102068 | 0.858 | 1.018 | 0.834 | 1.244 |
| ebi-a-GCST90018862 | finn-b-G6_MIGRAINE_NO_AURA | Migraine without aura \|\| id:finn-b-G6_MIGRAINE_NO_AURA | Hypothyroidism \|\| id:ebi-a-GCST90018862 | Weighted mode | 68 | 0.028775 | 0.059948 | 0.633 | 1.029 | 0.915 | 1.158 |
| finn-b-AUTOIMMUNE_HYPERTHYROIDISM | finn-b-G6_MIGRAINE_WITH_AURA | Migraine with aura \|\| id:finn-b-G6_MIGRAINE_WITH_AURA | Autoimmune hyperthyroidism \|\| id:finn-b-AUTOIMMUNE_HYPERTHYROIDISM | MR Egger | 7 | 0.093306 | 0.061874 | 0.192 | 1.098 | 0.972 | 1.239 |
| finn-b-AUTOIMMUNE_HYPERTHYROIDISM | finn-b-G6_MIGRAINE_WITH_AURA | Migraine with aura \|\| id:finn-b-G6_MIGRAINE_WITH_AURA | Autoimmune hyperthyroidism \|\| id:finn-b-AUTOIMMUNE_HYPERTHYROIDISM | Weighted median | 7 | 0.064591 | 0.03136 | 0.039 | 1.067 | 1.003 | 1.134 |
| finn-b-AUTOIMMUNE_HYPERTHYROIDISM | finn-b-G6_MIGRAINE_WITH_AURA | Migraine with aura \|\| id:finn-b-G6_MIGRAINE_WITH_AURA | Autoimmune hyperthyroidism \|\| id:finn-b-AUTOIMMUNE_HYPERTHYROIDISM | Inverse variance weighted | 7 | 0.071829 | 0.02625 | 0.006 | 1.074 | 1.021 | 1.131 |
| finn-b-AUTOIMMUNE_HYPERTHYROIDISM | finn-b-G6_MIGRAINE_WITH_AURA | Migraine with aura \|\| id:finn-b-G6_MIGRAINE_WITH_AURA | Autoimmune hyperthyroidism \|\| id:finn-b-AUTOIMMUNE_HYPERTHYROIDISM | Simple mode | 7 | 0.059089 | 0.041832 | 0.207 | 1.061 | 0.977 | 1.152 |
| finn-b-AUTOIMMUNE_HYPERTHYROIDISM | finn-b-G6_MIGRAINE_WITH_AURA | Migraine with aura \|\| id:finn-b-G6_MIGRAINE_WITH_AURA | Autoimmune hyperthyroidism \|\| id:finn-b-AUTOIMMUNE_HYPERTHYROIDISM | Weighted mode | 7 | 0.060635 | 0.041394 | 0.193 | 1.063 | 0.980 | 1.152 |
| finn-b-AUTOIMMUNE_HYPERTHYROIDISM | finn-b-G6_MIGRAINE_NO_AURA | Migraine without aura \|\| id:finn-b-G6_MIGRAINE_NO_AURA | Autoimmune hyperthyroidism \|\| id:finn-b-AUTOIMMUNE_HYPERTHYROIDISM | MR Egger | 7 | 0.049915 | 0.064964 | 0.477 | 1.051 | 0.926 | 1.194 |
| finn-b-AUTOIMMUNE_HYPERTHYROIDISM | finn-b-G6_MIGRAINE_NO_AURA | Migraine without aura \|\| id:finn-b-G6_MIGRAINE_NO_AURA | Autoimmune hyperthyroidism \|\| id:finn-b-AUTOIMMUNE_HYPERTHYROIDISM | Weighted median | 7 | 0.050936 | 0.034764 | 0.143 | 1.052 | 0.983 | 1.126 |
| finn-b-AUTOIMMUNE_HYPERTHYROIDISM | finn-b-G6_MIGRAINE_NO_AURA | Migraine without aura \|\| id:finn-b-G6_MIGRAINE_NO_AURA | Autoimmune hyperthyroidism \|\| id:finn-b-AUTOIMMUNE_HYPERTHYROIDISM | Inverse variance weighted | 7 | 0.065702 | 0.027569 | 0.017 | 1.068 | 1.012 | 1.127 |
| finn-b-AUTOIMMUNE_HYPERTHYROIDISM | finn-b-G6_MIGRAINE_NO_AURA | Migraine without aura \|\| id:finn-b-G6_MIGRAINE_NO_AURA | Autoimmune hyperthyroidism \|\| id:finn-b-AUTOIMMUNE_HYPERTHYROIDISM | Simple mode | 7 | 0.061054 | 0.041787 | 0.194 | 1.063 | 0.979 | 1.154 |
| finn-b-AUTOIMMUNE_HYPERTHYROIDISM | finn-b-G6_MIGRAINE_NO_AURA | Migraine without aura \|\| id:finn-b-G6_MIGRAINE_NO_AURA | Autoimmune hyperthyroidism \|\| id:finn-b-AUTOIMMUNE_HYPERTHYROIDISM | Weighted mode | 7 | 0.053027 | 0.041465 | 0.248 | 1.054 | 0.972 | 1.144 |
| ebi-a-GCST003156 | finn-b-G6_MIGRAINE_WITH_AURA | Migraine with aura \|\| id:finn-b-G6_MIGRAINE_WITH_AURA | Systemic lupus erythematosus \|\| id:ebi-a-GCST003156 | MR Egger | 42 | 0.072255 | 0.031821 | 0.029 | 1.075 | 1.010 | 1.144 |
| ebi-a-GCST003156 | finn-b-G6_MIGRAINE_WITH_AURA | Migraine with aura \|\| id:finn-b-G6_MIGRAINE_WITH_AURA | Systemic lupus erythematosus \|\| id:ebi-a-GCST003156 | Weighted median | 42 | 0.050607 | 0.02183 | 0.020 | 1.052 | 1.008 | 1.098 |
| ebi-a-GCST003156 | finn-b-G6_MIGRAINE_WITH_AURA | Migraine with aura \|\| id:finn-b-G6_MIGRAINE_WITH_AURA | Systemic lupus erythematosus \|\| id:ebi-a-GCST003156 | Inverse variance weighted | 42 | 0.050317 | 0.014681 | 0.001 | 1.052 | 1.022 | 1.082 |
| ebi-a-GCST003156 | finn-b-G6_MIGRAINE_WITH_AURA | Migraine with aura \|\| id:finn-b-G6_MIGRAINE_WITH_AURA | Systemic lupus erythematosus \|\| id:ebi-a-GCST003156 | Simple mode | 42 | 0.057984 | 0.038133 | 0.136 | 1.060 | 0.983 | 1.142 |
| ebi-a-GCST003156 | finn-b-G6_MIGRAINE_WITH_AURA | Migraine with aura \|\| id:finn-b-G6_MIGRAINE_WITH_AURA | Systemic lupus erythematosus \|\| id:ebi-a-GCST003156 | Weighted mode | 42 | 0.052716 | 0.030114 | 0.088 | 1.054 | 0.994 | 1.118 |
| ebi-a-GCST003156 | finn-b-G6_MIGRAINE_NO_AURA | Migraine without aura \|\| id:finn-b-G6_MIGRAINE_NO_AURA | Systemic lupus erythematosus \|\| id:ebi-a-GCST003156 | MR Egger | 42 | 0.014867 | 0.033507 | 0.660 | 1.015 | 0.950 | 1.084 |
| ebi-a-GCST003156 | finn-b-G6_MIGRAINE_NO_AURA | Migraine without aura \|\| id:finn-b-G6_MIGRAINE_NO_AURA | Systemic lupus erythematosus \|\| id:ebi-a-GCST003156 | Weighted median | 42 | -0.01667 | 0.022929 | 0.467 | 0.983 | 0.940 | 1.029 |
| ebi-a-GCST003156 | finn-b-G6_MIGRAINE_NO_AURA | Migraine without aura \|\| id:finn-b-G6_MIGRAINE_NO_AURA | Systemic lupus erythematosus \|\| id:ebi-a-GCST003156 | Inverse variance weighted | 42 | -0.01532 | 0.015466 | 0.322 | 0.985 | 0.955 | 1.015 |
| ebi-a-GCST003156 | finn-b-G6_MIGRAINE_NO_AURA | Migraine without aura \|\| id:finn-b-G6_MIGRAINE_NO_AURA | Systemic lupus erythematosus \|\| id:ebi-a-GCST003156 | Simple mode | 42 | -0.03234 | 0.046373 | 0.489 | 0.968 | 0.884 | 1.060 |
| ebi-a-GCST003156 | finn-b-G6_MIGRAINE_NO_AURA | Migraine without aura \|\| id:finn-b-G6_MIGRAINE_NO_AURA | Systemic lupus erythematosus \|\| id:ebi-a-GCST003156 | Weighted mode | 42 | -0.0103 | 0.031121 | 0.742 | 0.990 | 0.931 | 1.052 |
| ebi-a-GCST005529 | finn-b-G6_MIGRAINE_WITH_AURA | Migraine with aura \|\| id:finn-b-G6_MIGRAINE_WITH_AURA | Ankylosing spondylitis \|\| id:ebi-a-GCST005529 | MR Egger | 24 | -0.11837 | 0.193502 | 0.547 | 0.888 | 0.608 | 1.298 |
| ebi-a-GCST005529 | finn-b-G6_MIGRAINE_WITH_AURA | Migraine with aura \|\| id:finn-b-G6_MIGRAINE_WITH_AURA | Ankylosing spondylitis \|\| id:ebi-a-GCST005529 | Weighted median | 24 | 0.080122 | 0.158265 | 0.613 | 1.083 | 0.794 | 1.477 |
| ebi-a-GCST005529 | finn-b-G6_MIGRAINE_WITH_AURA | Migraine with aura \|\| id:finn-b-G6_MIGRAINE_WITH_AURA | Ankylosing spondylitis \|\| id:ebi-a-GCST005529 | Inverse variance weighted | 24 | -0.10249 | 0.112032 | 0.360 | 0.903 | 0.725 | 1.124 |
| ebi-a-GCST005529 | finn-b-G6_MIGRAINE_WITH_AURA | Migraine with aura \|\| id:finn-b-G6_MIGRAINE_WITH_AURA | Ankylosing spondylitis \|\| id:ebi-a-GCST005529 | Simple mode | 24 | -0.33404 | 0.287346 | 0.257 | 0.716 | 0.408 | 1.258 |
| ebi-a-GCST005529 | finn-b-G6_MIGRAINE_WITH_AURA | Migraine with aura \|\| id:finn-b-G6_MIGRAINE_WITH_AURA | Ankylosing spondylitis \|\| id:ebi-a-GCST005529 | Weighted mode | 24 | 0.004467 | 0.168957 | 0.979 | 1.004 | 0.721 | 1.399 |
| ebi-a-GCST005529 | finn-b-G6_MIGRAINE_NO_AURA | Migraine without aura \|\| id:finn-b-G6_MIGRAINE_NO_AURA | Ankylosing spondylitis \|\| id:ebi-a-GCST005529 | MR Egger | 24 | -0.0991 | 0.219344 | 0.656 | 0.906 | 0.589 | 1.392 |
| ebi-a-GCST005529 | finn-b-G6_MIGRAINE_NO_AURA | Migraine without aura \|\| id:finn-b-G6_MIGRAINE_NO_AURA | Ankylosing spondylitis \|\| id:ebi-a-GCST005529 | Weighted median | 24 | -0.09604 | 0.163179 | 0.556 | 0.908 | 0.660 | 1.251 |
| ebi-a-GCST005529 | finn-b-G6_MIGRAINE_NO_AURA | Migraine without aura \|\| id:finn-b-G6_MIGRAINE_NO_AURA | Ankylosing spondylitis \|\| id:ebi-a-GCST005529 | Inverse variance weighted | 24 | -0.08448 | 0.124255 | 0.497 | 0.919 | 0.720 | 1.172 |
| ebi-a-GCST005529 | finn-b-G6_MIGRAINE_NO_AURA | Migraine without aura \|\| id:finn-b-G6_MIGRAINE_NO_AURA | Ankylosing spondylitis \|\| id:ebi-a-GCST005529 | Simple mode | 24 | 0.682452 | 0.355529 | 0.067 | 1.979 | 0.986 | 3.972 |
| ebi-a-GCST005529 | finn-b-G6_MIGRAINE_NO_AURA | Migraine without aura \|\| id:finn-b-G6_MIGRAINE_NO_AURA | Ankylosing spondylitis \|\| id:ebi-a-GCST005529 | Weighted mode | 24 | -0.13484 | 0.161822 | 0.413 | 0.874 | 0.636 | 1.200 |

**Supplementary Table 5 Analyzing the relationship between genetic prediction of migraine and autoimmune diseases by various MR analyses.**

| id.exposure | id.outcome | outcome | exposure | method | nsnp | b | se | pval | or | or_lci95 | or_uci95 |
| --- | --- | --- | --- | --- | --- | --- | --- | --- | --- | --- | --- |
| finn-b-G6_MIGRAINE | finn-b-AUTOIMMUNE_HYPERTHYROIDISM | Autoimmune hyperthyroidism \|\| id:finn-b-AUTOIMMUNE_HYPERTHYROIDISM | \|\| id:finn-b-G6_MIGRAINE | MR Egger | 13 | -0.01005 | 0.450269 | 0.982601 | 0.990005 | 0.409601 | 2.392841 |
| finn-b-G6_MIGRAINE | finn-b-AUTOIMMUNE_HYPERTHYROIDISM | Autoimmune hyperthyroidism \|\| id:finn-b-AUTOIMMUNE_HYPERTHYROIDISM | \|\| id:finn-b-G6_MIGRAINE | Weighted median | 13 | 0.21743 | 0.212944 | 0.307224 | 1.242878 | 0.818779 | 1.886646 |
| finn-b-G6_MIGRAINE | finn-b-AUTOIMMUNE_HYPERTHYROIDISM | Autoimmune hyperthyroidism \|\| id:finn-b-AUTOIMMUNE_HYPERTHYROIDISM | \|\| id:finn-b-G6_MIGRAINE | Inverse variance weighted | 13 | 0.299708 | 0.182806 | 0.101112 | 1.349465 | 0.943092 | 1.930942 |
| finn-b-G6_MIGRAINE | finn-b-AUTOIMMUNE_HYPERTHYROIDISM | Autoimmune hyperthyroidism \|\| id:finn-b-AUTOIMMUNE_HYPERTHYROIDISM | \|\| id:finn-b-G6_MIGRAINE | Simple mode | 13 | 0.228939 | 0.315185 | 0.481544 | 1.257266 | 0.677854 | 2.331943 |
| finn-b-G6_MIGRAINE | finn-b-AUTOIMMUNE_HYPERTHYROIDISM | Autoimmune hyperthyroidism \|\| id:finn-b-AUTOIMMUNE_HYPERTHYROIDISM | \|\| id:finn-b-G6_MIGRAINE | Weighted mode | 13 | 0.220547 | 0.301255 | 0.478166 | 1.246759 | 0.690795 | 2.250173 |
| finn-b-G6_MIGRAINE_WITH_AURA | finn-b-AUTOIMMUNE_HYPERTHYROIDISM | Autoimmune hyperthyroidism \|\| id:finn-b-AUTOIMMUNE_HYPERTHYROIDISM | \|\| id:finn-b-G6_MIGRAINE_WITH_AURA | MR Egger | 9 | 0.015852 | 0.291021 | 0.958083 | 1.015978 | 0.574331 | 1.797242 |
| finn-b-G6_MIGRAINE_WITH_AURA | finn-b-AUTOIMMUNE_HYPERTHYROIDISM | Autoimmune hyperthyroidism \|\| id:finn-b-AUTOIMMUNE_HYPERTHYROIDISM | \|\| id:finn-b-G6_MIGRAINE_WITH_AURA | Weighted median | 9 | 0.108695 | 0.174703 | 0.533828 | 1.114823 | 0.791582 | 1.570058 |
| finn-b-G6_MIGRAINE_WITH_AURA | finn-b-AUTOIMMUNE_HYPERTHYROIDISM | Autoimmune hyperthyroidism \|\| id:finn-b-AUTOIMMUNE_HYPERTHYROIDISM | \|\| id:finn-b-G6_MIGRAINE_WITH_AURA | Inverse variance weighted | 9 | 0.06344 | 0.125908 | 0.614359 | 1.065496 | 0.832485 | 1.363726 |
| finn-b-G6_MIGRAINE_WITH_AURA | finn-b-AUTOIMMUNE_HYPERTHYROIDISM | Autoimmune hyperthyroidism \|\| id:finn-b-AUTOIMMUNE_HYPERTHYROIDISM | \|\| id:finn-b-G6_MIGRAINE_WITH_AURA | Simple mode | 9 | -0.00718 | 0.263202 | 0.978918 | 0.99285 | 0.592709 | 1.663127 |
| finn-b-G6_MIGRAINE_WITH_AURA | finn-b-AUTOIMMUNE_HYPERTHYROIDISM | Autoimmune hyperthyroidism \|\| id:finn-b-AUTOIMMUNE_HYPERTHYROIDISM | \|\| id:finn-b-G6_MIGRAINE_WITH_AURA | Weighted mode | 9 | 0.010848 | 0.271965 | 0.969159 | 1.010907 | 0.593212 | 1.722711 |
| finn-b-G6_MIGRAINE_NO_AURA | finn-b-AUTOIMMUNE_HYPERTHYROIDISM | Autoimmune hyperthyroidism \|\| id:finn-b-AUTOIMMUNE_HYPERTHYROIDISM | \|\| id:finn-b-G6_MIGRAINE_NO_AURA | MR Egger | 10 | -0.38808 | 0.274464 | 0.195085 | 0.678356 | 0.396123 | 1.161678 |
| finn-b-G6_MIGRAINE_NO_AURA | finn-b-AUTOIMMUNE_HYPERTHYROIDISM | Autoimmune hyperthyroidism \|\| id:finn-b-AUTOIMMUNE_HYPERTHYROIDISM | \|\| id:finn-b-G6_MIGRAINE_NO_AURA | Weighted median | 10 | -0.1618 | 0.160452 | 0.31327 | 0.850614 | 0.621088 | 1.164962 |
| finn-b-G6_MIGRAINE_NO_AURA | finn-b-AUTOIMMUNE_HYPERTHYROIDISM | Autoimmune hyperthyroidism \|\| id:finn-b-AUTOIMMUNE_HYPERTHYROIDISM | \|\| id:finn-b-G6_MIGRAINE_NO_AURA | Inverse variance weighted | 10 | -0.21595 | 0.123258 | 0.079771 | 0.805775 | 0.632841 | 1.025967 |
| finn-b-G6_MIGRAINE_NO_AURA | finn-b-AUTOIMMUNE_HYPERTHYROIDISM | Autoimmune hyperthyroidism \|\| id:finn-b-AUTOIMMUNE_HYPERTHYROIDISM | \|\| id:finn-b-G6_MIGRAINE_NO_AURA | Simple mode | 10 | -0.43971 | 0.296889 | 0.17273 | 0.644224 | 0.360014 | 1.1528 |
| finn-b-G6_MIGRAINE_NO_AURA | finn-b-AUTOIMMUNE_HYPERTHYROIDISM | Autoimmune hyperthyroidism \|\| id:finn-b-AUTOIMMUNE_HYPERTHYROIDISM | \|\| id:finn-b-G6_MIGRAINE_NO_AURA | Weighted mode | 10 | -0.43971 | 0.289708 | 0.163386 | 0.644224 | 0.365117 | 1.136688 |
| finn-b-G6_MIGRAINE_NO_AURA | ebi-a-GCST90018862 | Hypothyroidism \|\| id:ebi-a-GCST90018862 | \|\| id:finn-b-G6_MIGRAINE_NO_AURA | MR Egger | 10 | 0.003803 | 0.053395 | 0.94497 | 1.00381 | 0.904067 | 1.114558 |
| finn-b-G6_MIGRAINE_NO_AURA | ebi-a-GCST90018862 | Hypothyroidism \|\| id:ebi-a-GCST90018862 | \|\| id:finn-b-G6_MIGRAINE_NO_AURA | Weighted median | 10 | 0.041695 | 0.029947 | 0.163837 | 1.042577 | 0.983142 | 1.105604 |
| finn-b-G6_MIGRAINE_NO_AURA | ebi-a-GCST90018862 | Hypothyroidism \|\| id:ebi-a-GCST90018862 | \|\| id:finn-b-G6_MIGRAINE_NO_AURA | Inverse variance weighted | 10 | 0.029351 | 0.028107 | 0.296378 | 1.029786 | 0.974589 | 1.088109 |
| finn-b-G6_MIGRAINE_NO_AURA | ebi-a-GCST90018862 | Hypothyroidism \|\| id:ebi-a-GCST90018862 | \|\| id:finn-b-G6_MIGRAINE_NO_AURA | Simple mode | 10 | 0.049058 | 0.048176 | 0.335125 | 1.050281 | 0.955647 | 1.154287 |
| finn-b-G6_MIGRAINE_NO_AURA | ebi-a-GCST90018862 | Hypothyroidism \|\| id:ebi-a-GCST90018862 | \|\| id:finn-b-G6_MIGRAINE_NO_AURA | Weighted mode | 10 | 0.025495 | 0.041057 | 0.550023 | 1.025823 | 0.946507 | 1.111785 |
| finn-b-G6_MIGRAINE_WITH_AURA | ebi-a-GCST003156 | Systemic lupus erythematosus \|\| id:ebi-a-GCST003156 | \|\| id:finn-b-G6_MIGRAINE_WITH_AURA | MR Egger | 7 | 0.175336 | 0.148745 | 0.291526 | 1.191646 | 0.890293 | 1.595004 |
| finn-b-G6_MIGRAINE_WITH_AURA | ebi-a-GCST003156 | Systemic lupus erythematosus \|\| id:ebi-a-GCST003156 | \|\| id:finn-b-G6_MIGRAINE_WITH_AURA | Weighted median | 7 | 0.105053 | 0.098146 | 0.284451 | 1.110769 | 0.91639 | 1.346379 |
| finn-b-G6_MIGRAINE_WITH_AURA | ebi-a-GCST003156 | Systemic lupus erythematosus \|\| id:ebi-a-GCST003156 | \|\| id:finn-b-G6_MIGRAINE_WITH_AURA | Inverse variance weighted | 7 | 0.058601 | 0.074624 | 0.432291 | 1.060352 | 0.91607 | 1.227357 |
| finn-b-G6_MIGRAINE_WITH_AURA | ebi-a-GCST003156 | Systemic lupus erythematosus \|\| id:ebi-a-GCST003156 | \|\| id:finn-b-G6_MIGRAINE_WITH_AURA | Simple mode | 7 | 0.133924 | 0.130805 | 0.345402 | 1.143306 | 0.884747 | 1.477427 |
| finn-b-G6_MIGRAINE_WITH_AURA | ebi-a-GCST003156 | Systemic lupus erythematosus \|\| id:ebi-a-GCST003156 | \|\| id:finn-b-G6_MIGRAINE_WITH_AURA | Weighted mode | 7 | 0.12695 | 0.113094 | 0.304545 | 1.13536 | 0.909632 | 1.417103 |
